# Oxidative Stability of Virgin Avocado Oil Enriched with Avocado Leaves and Olive-Fruit-Processing By-Products (Leaves, Pomace) via Ultrasound-Assisted Maceration

**DOI:** 10.3390/foods14020294

**Published:** 2025-01-17

**Authors:** Ioanna Pyrka, Stavros Stefanidis, Stella A. Ordoudi, Sofia Lalou, Nikolaos Nenadis

**Affiliations:** 1 Laboratory of Food Chemistry and Technology, School of Chemistry, Aristotle University of Thessaloniki, 54124 Thessaloniki, Greece; ioannapyrka@chem.auth.gr (I.P.); stavstef@chem.auth.gr (S.S.); 2 Laboratory of Oenology and Alcoholic Beverages, Department of Food Science and Technology, School of Agriculture, Aristotle University of Thessaloniki, 54124 Thessaloniki, Greece; steord@agro.auth.gr; 3 Natural Products Research Centre of Excellence-AUTH (NatPro-AUTH), Center for Interdisciplinary Research and Innovation (CIRI-AUTH), 57001 Thessaloniki, Greece; 4 Department of Food Science and Technology, Perrotis College, American Farm School, 55102 Thessaloniki, Greece; slalou@afs.edu.gr

**Keywords:** virgin avocado oil, ultrasound-assisted maceration, oil enrichment, fortified oil, oxidative stability, avocado leaves, olive leaves, olive pomace, antioxidants

## Abstract

Virgin avocado oil (VAO), treasured for its nutritional and sensory properties, is susceptible to oxidation. To improve its oxidative stability, the feasibility of enrichment with antioxidants from avocado or olive-processing by-products via ultrasound-assisted maceration was explored. Dried, milled avocado (AL), olive leaves (OL), or olive pomace (OP) were ultrasound-macerated with laboratory-extracted VAO at 5, 10, and 20% *w/w* levels. Induction-period (IP) values, determined via Rancimat (110 °C, 20 L/h), increased by 1.1–1.6-fold. Maceration with AL and OL added pigments (β-carotene, lutein, α-chlorophyll, and α-pheophytin) but only AL significantly boosted α-tocopherol levels (up to 3.7-fold). Extraction of major polar phenols (chlorogenic acid, oleuropein, and hydroxytyrosol) was poor (<42 mg/kg oil). Oleanolic and maslinic acids, from OL and OP, reached up to 650 and 260 mg/kg. The IP values correlated (*r* = 0.796, *p* = 0.002) only with total polar phenol content. Maceration with OP resulted in superior antioxidant activity, extending the predicted shelf-life from 14 to 22.3 months, reaching that of a hydroxytyrosol-rich extra-virgin olive oil (24.9 months). GC-MS revealed the dominance of volatile acids in OL- and OP-VAOs, and estragole in AL-VAO highlighting some organoleptic and safety challenges to be considered, particularly when aiming to exploit these materials for the enhancement of the oxidative stability of VAOs to sustain its production.

## 1. Introduction

Recently, there has been a rising trend among European citizens in consuming minimally processed foods with health benefits and premium sensory properties. As a result, the demand for edible virgin oils (VOs) and cold-pressed oils (CPOs), which are rich sources of bioactive substances, has increased. Thus, particular attention is given to virgin avocado oil (VAO), which is characterized by highly attractive sensory characteristics—defined by butter-like, green aroma notes and the absence of pungency—together with the well-known health properties of the avocado fruit. The fatty acid composition of VAO resembles that of virgin olive oil (VOO) as it is rich in monounsaturated (mainly oleic acid) rather than polyunsaturated fatty acids. Sterols and other liposoluble bioactive compounds such as *α*-tocopherol and carotenoids are also found in virgin, unrefined avocado oil. On the other hand, low-molecular-weight, polar phenolic compounds, known as efficient free-radical scavengers, are usually absent [1]. Several studies support this evidence, although the phenol composition data from chromatographic analyses are rather scarce [2,3,4]. The naturally poor content in polar phenols is probably the reason why VAO is highly susceptible to oxidation and displays a rather short shelf-life [2] (e.g., 7.06 h, 110 °C, 20 L/h). Thus, there is a need to improve the oxidative stability of VAOs while maintaining their functional properties over a longer storage period. Recently, Flores et al. [5] proposed the addition of an extract from an endemic Chilean berry (maqui) by-product (leaves) to improve the thermal stability of the oil. To our knowledge, this is the only report on avocado oil treatment for such a purpose.

Nowadays, the sustainability of the avocado oil industry is an issue for both producers and consumers, considering the global distribution channels and the seasonality of the market [6]. In the Mediterranean areas where avocado cultivation was introduced a few decades ago, there is still a gap in knowledge about the optimum oil-processing technologies and quality specifications. Nevertheless, short supply chains are in favor of premium quality products. In addition to that, the circular economy concept that gained popularity over the last few years promotes the idea of reusing local agro-industrial by-products and upcycling by-products as carriers of bioactive constituents or fortification agents of edible oils [1]. Such a trend applies to the production and trade of minimally processed, high-quality VO and CPO products. Avocado tree by-products and residues, e.g., leaves, peels, and seeds, have never been used for this particular purpose despite their potential as sources of antioxidant ingredients [7]. Instead, in a work published a few years ago, avocado and olive-leaf extracts were used to enhance the thermal stability of canola and high-oleic sunflower oils during frying [8]. The authors reported that under thermal oxidation conditions, B-type trimer procyanidins, as the major phenolic compounds in polar avocado leaf extracts, were less effective than oleuropein, the major active constituent of olive-leaf extract; this performance was attributed to well-established structure–activity relationships. In the same prospect, a dried pomace obtained from a two-phase olive mill—particularly a pulp-rich fraction (OP) containing an appreciable amount of hydroxytyrosol and triterpenic acids [9]—could be a candidate material for fortification with antioxidants. OP powders have been proposed as a safe source of bioactives for their direct addition to foods and as a sustainable approach instead of extracts [10].

Therefore, considering the above, the present study aimed to explore the feasibility of using avocado leaves, olive leaves, and olive pomace to improve the shelf-life and stability of VAO under autoxidation conditions.

## 2. Materials and Methods

### 2.1. Chemicals and Standards

Gallic acid, quercetin, Trolox (6-hydroxy-2,5,7,8-tetramethyl-chroman-2-carboxylic acid), phosphoric acid, DPPH^●^ (1,1-diphenyl-2-picrylhydrazyl radical), neocuproine, and α-chlorophyll were purchased from Sigma Chemical Co. (St. Louis and Burlington, MA, USA). Oleuropein, luteolin-7-O-glucoside, and lutein were obtained from Extrasynthese S.A. (Genay, France), whereas maslinic and oleanolic acids from Carbosynth Ltd. (Compton, UK). Methanol, ethanol, water, methyl tert-butyl ether (MTBE), sodium carbonate, sodium hydroxide, Folin–Ciocalteu reagent, and acetic acid were acquired from Chem-Lab NV (Zedelgem, Belgium); acetonitrile, isopropanol, and potassium iodide were obtained from Honeywell (Charlotte, NC, USA); aluminum chloride from Fluka Chemie (Buchs, Switzerland); β-carotene, ammonium acetate, sodium thiosulfate, and copper chloride from Merck KGaA (Darmstadt, Germany); iso-octane and n-hexane from VWR Chemicals BDH (Radnor, PA, USA); diethyl-ether from Panreac Quimica (Barcelona, Spain).

### 2.2. Plant Materials

Avocado fruits (*Persea americana* Mill., cv. Fuerte) were purchased from a trusted wholesale supplier of Cretan fruits during January 2023 (approximately 25 kg). The fruits were left at room temperature until they reached the “ready to eat” stage of ripeness (10 days).

Mature avocado leaves (AL) were sampled upon request, from cv. Fuerte avocado trees that are grown at the Institute of Olive, Subtropical Plants, and Vine in Crete (Chania, Greece). The samples were collected on 28 September 2022 and shipped to the laboratory within two days. The fresh material was split into 10 portions of approximately 100 g intact leaves, packed into vacuum bags, and stored in the freezer (−40 °C) until use. Before treatments, the frozen leaves were defrosted, cut into small pieces, split into 20 g lots, and dried in a domestic microwave oven (K30GMS18E, 1400 W) for 2 min (2 *×* 1 min) (conditions found after preliminary trials). Olive (*Olea europaea* L., cv. Koroneiki) leaves (OL) were harvested in May 2023 from different trees grown in the orchard of Perrotis College (Thessaloniki, Greece). The leaves were dried using intermittent near-infrared radiation in a Toshiba TL1-AC25CZA(BS) digital toaster oven, according to previously optimized conditions [11] (thin layer, 116 °C, 3 min). Dried AL and OL samples were milled and sieved to obtain powders with less than a 500-micrometer particle size. Dried olive pomace (OP) (cv. Koroneiki) was obtained from the Union of Agricultural Cooperative of Lakonia (Greece). A pulp-rich fraction (≤500 μm), previously dried at 140 °C to reach a high hydroxytyrosol content [9] (3.2 g/kg dw), was used for the fortification experiments.

For compositional analyses, extraction was carried out during a 30-minute maceration time in an ultrasound bath (P 30H, Elmasonic, Elma) using a mixture of EtOH:H_2_O, 1:1, *v/v* (AL), or MeOH (OL and OP), and varying feed-to-solvent ratios: 10:100 (AL), 2:100 (OL), 5:100 (OP). The temperature was kept constantly below 35 °C (conditions found after preliminary trials), 60 °C [12], or 30 °C [9] in each of the experiments, respectively. The extracts were characterized regarding their total phenolic and flavonoid contents, antioxidant activity, and the content of individual phenolic and triterpene compounds, as described below.

### 2.3. Virgin Avocado Oil Extraction

Virgin avocado oil was used as a base for the fortification treatments (C-VAO) and was laboratory-extracted using an Abencor system with the conditions recommended by Green and Wang [3] without the addition of talc, enzyme, and water. In brief, avocado fruits were washed, de-stoned, and homogenized with a blender to obtain pulp (≈20 kg) that was subsequently transferred to an Abencor thermo beater (THERMO-MIXER TB-100, MC2 Ingeniería Y Sistemas SL, Sevilla, Spain) and malaxated for 3 h at ≤45 °C. The sample was centrifuged to obtain the light green oil (approximately 10% *v/w*) that was then stored without headspace at 4 °C until use.

### 2.4. VAO Fortification Treatments

AL, OL, and OP powder samples were macerated with C-VAO using three addition levels on a weight basis: 5% (AL5, OL5, OP5), 10% (AL10, OL10, OP10), and 20% *w/w* (AL20, OL20, OP20). Each mixture was macerated for 30 min using a mechanical stirrer set at 150 rpm (IKA Labortechnik RW 20.n) and subsequently exposed to ultrasound using a bath at ≤30 °C (Elmasonic S 30 H, Elma Schmidbauer GmbH, Singen, Germany; 37 kHz, 80 W, capacity: 2.75 L). The solid parts were then removed by centrifugation (ST16R Refrigerated Centrifuge, Thermo Scientific, Waltham, MA, USA) at 10,000× *g* for 10 min and the supernatant oils were stored in 6 mL bottles without headspace at −20 °C until analysis. Each sample was prepared in triplicate.

For comparison purposes, two reference oil products from the collection of the LFCT were used as follows: a freshly produced avocado oil (C-AO) that was donated by Evercrete–Antonakis Bros (Chania, Greece); and an extra virgin olive oil (C-ΕVOO), labeled as “early harvest”, that was donated by Terra Creta (Chania, Greece).

### 2.5. Attenuated Total Reflectance—Fourier-Transform Infrared (ATR-FTIR) Spectroscopy

FTIR spectra of oil samples (0.8 mL) were obtained using an IRAffinity-1 spectrometer (Shimadzu Fourier-Transform Infrared Spectrophotometer, Shimadzu Corp., Kyoto, Japan) with the aid of a horizontal attenuated total reflectance (ATR) cell. Spectra were recorded in the range of 650–4000 cm^−1^ at a resolution of 4 cm^−1^ by co-adding 64 interferograms. Each absorbance spectrum was collected against a background obtained with a dry and empty ATR cell. Air and ATR correction at 650 cm^−1^ were calculated using IR solution software (version 1.50, Shimadzu Corporation, Kyoto, Japan). The spectra were then smoothed (11 p, 2nd order, Savitzky–Golay).

### 2.6. Basic Quality Parameters of Oils

Free fatty acid (FFA) content expressed as oleic acid (%), peroxide value (PV) expressed as meq O_2_/kg oil, and K_232_ and K_270_ extinction coefficients were determined titrimetrically (FFA, PV) and spectroscopically (K_232/270_) following the analytical methods of AOCS Ca 5a-40, Cd 8b-90, and Ch 5-91, respectively [13]. Regarding K_232_ and K_270_—which express the content in conjugated diene and triene products of polyunsaturated fatty acid oxidation—calculation was carried with the following equations, K_232_ = A_232_/*c* × *d* and K_270_ = A_270_/*c* × *d*, where A_232_ and A_270_ are the UV absorbance values recorded at 232 and 270 nm, respectively; *c* is the concentration of the oil solution in iso-octane expressed as % *w/v* (a 0.025 g oil sample was weighted and diluted to 10 mL iso-octane); and *d* is the cell thickness in cm (1 cm).

### 2.7. Oxidative Stability of Oils

Oxidative stability was assessed by determination of the induction period (in hours) using the Rancimat test (model 892, Metrohm Co., Basel, Switzerland), employing 3 g of oil heated to 110 °C with a stable air flow of 20 L/h. A rough estimate of the shelf-life (in months) of the enriched products at room temperature was carried out by the empirical Q10 approach, as described by the manufacturer (https://www.metrohm.com/en/products/2/8920/28920010.html, accessed on 23 January 2024). The protection factor of the enriched oils, which is also an indication of oxidative stability, was also calculated according to Bersuder and Smith [14]: protection Factor = induction period of enriched oil / induction period of control (C-VAO).

### 2.8. Antioxidant Activity (AA) Assays

AA was estimated (i) on oil diluted in isopropanol (10% *w/v*) using the DPPH^●^ assay, and (ii) on the polar fractions of the oils and the extracts of AL, OL, and OP using both DPPH^●^ and CUPRAC assays. The polar fractions of the oils were extracted using the protocol of Papoti and Tsimidou [15] with minor modifications. Briefly, 2 g of oil sample was diluted in 5 mL of hexane and 5 mL of MeOH/H_2_O (60/40 *v/v*) was added. The mixture was vortexed for 2 min and the polar and non-polar fractions were separated after centrifugation at 700× *g* for 10 min.

DPPH^●^ was applied according to Nenadis and Tsimidou [16] at 516 nm using 0.25–1 mL of diluted oil and 150 or 300 μL of the polar fraction of oil; CUPRAC was applied according to Apak et al. [17], recording the absorbance at 450 nm, using the same volumes of diluted oil. AA assays were also applied to the extracts of the enrichment agents, using 30 μL of AL (1:20 dilution) and 20 μL of OL and OP extracts. Results are expressed as Trolox equivalents (mmol TE/kg oil and mol TE/kg dw).

### 2.9. Chemical Composition Analyses

#### 2.9.1. UV–Vis-Based Assays

##### Total Phenolic Content (TPC) and Total Flavonoid Content (TFC)

TPC was determined by the Folin–Ciocalteu assay, recording the absorbance at 725 nm. A total of 400 μL of the oils’ polar fraction or 60 μL from a 1:20 dilution of AL extract, 40 μL of OL extract, and 70 μL of OP extract were used. Results are expressed as gallic-acid equivalents per kg of oil or kg of dry weight of AL, OL, and OP (mg GAE/kg oil, g GAE/kg dw).

TFC was determined for AL, OL, and OP extracts by the ALCl3 method of Cvek et al. [18], recording the absorbance at 415 nm, using 30 μL, 300 μL, and 800 μL, respectively. Results are expressed as quercetin equivalents (g QUE/kg dw).

##### Total Chlorophyll and Carotenoid Contents

For the spectrophotometric analysis of total chlorophylls and carotenoids, the oils were diluted in hexane/isopropanol 99:1 (8% *w/w* final concentration) and examined using a UV-1601 UV–Vis Spectrophotometer (Shimadzu, Kyoto, Japan). The method was based on the protocol described by Minguez-Mosquera et al. [19] with the following formulas, total chlorophylls (mg pheophytin/kg oil) = (A_670_ × 106)/613 × 100 × *d*) and total carotenoids (mg lutein/kg oil) = (A_470_ × 106)/(2000 × 100 × *d*), where A_670_ and A_470_ are the absorbance values at 670 and 470 nm, respectively; 613 and 2000 are the extinction coefficients of pheophytin and lutein, respectively; and *d* is the cell thickness in cm (1 cm).

#### 2.9.2. GC-FID Analysis

##### Fatty Acid Composition

Fatty acid methyl esters were determined according to European Commission Regulation No. 2568/91 [20] with minor modifications. The fatty acids of the oil samples were converted to their methyl esters as follows: A total of 0.1 g of oil was diluted in 2 mL hexane and shaken for 1 min. Afterward, 0.2 mL of methanolic KOH was added and shaken for 1 min, a small amount of anhydrous Na_2_SO_4_ was added, and the mixture was left for 5 min. The supernatant phase was used for the analysis. The separation of different fatty acid methyl esters was performed by gas chromatography coupled to a flame ionization detector on a DB-23 (30 m × 0.32 μm × 0.25 μm) capillary column (Agilent J&W, Palo Alto, CA, USA) under the following conditions: carrier gas, hydrogen; flow rate, 1.1 mL/min; column temperature gradient, 100 °C (5 min), 100–240 °C (20 min), 240 °C (60 min); injection system and detector temperature, 240 °C; injection volume, 2 μL (split 50:1). Identification of the fatty acids was carried out by comparison of their retention times with those of standard fatty acid methyl esters (C4–C24). Results for each fatty acid are expressed as follows: peak ratio (%) = (peak area of individual fatty acid/total peak area of identified fatty acids) × 100. The % values were used to determine the oxidative susceptibility (OS) according to the following formula: OS = monounsaturated fatty acids (MUFAs) + (45 × C18:2) + (100 × C18:3) [21].

#### 2.9.3. HPLC-Based Assays

##### Identification and Quantification of Phenolic Compounds

Identification of phenolics and flavonoids of avocado leaf extract was performed using a quadrupole Orbitrap mass spectrometer (LTQ OrbiTrap MS) equipped with a heated electrospray ionization probe (HESI-II; ThermoFisher Scientific, Bremen, Germany). The mass spectrometer operated in negative-ion mode and MS spectra were acquired by full-range acquisition covering 50–1500 *m/z*. The HESI parameters were optimized as follows: sheath gas flow rate, 20 arbitrary units; auxiliary gas unit flow rate, 2 arbitrary units; capillary temperature, 270 °C; capillary voltage, −35 V; spray voltage, 4.2 kV; tube lens voltage, 50 V. Concentration of avocado leaf extract was 6 ppm in methanol and directly infused in the spectrometer with a flow rate of 10 μL/min. Phenol identification of olive leaves and olive pomace extracts was carried out using an HPLC-MS as previously described by Pyrka et al. [11].

Quantification of selected phenolic compounds was carried out in a Nexera X2 UHPLC system (Shimadzu, Kyoto, Japan), consisting of an SIL-30 AC autosampler, LC-30b CE pump, CTO-20 AC oven, SPD-M20A diode array, and RF-20AXS fluorescence detector, using an Accucore C18, 100 mm, 4.6 mm, 2.6 μm (Thermo Scientific, San Jose, CA, USA). The mobile phase consisted of (A) water acidified with 0.2% H_3_PO_4_; and (B) acetonitrile. The gradient elution protocol was as follows: 0 min, 10% B; 4 min, 20% B; 17.2 min, 50% B; 19.2, 95% B; 20.8 min, 95% B; 24 min, 10% B. The total run was 30 min. The flow rate was set at 0.5 mL/min. The injection volumes were 10 μL of the oils’ polar fraction or 1 μL of AL extract (1:2 dilution), 2 μL of OL extract, and 5 μL of OP extract. Chlorogenic acid was quantified at 330 nm; oleuropein, hydroxytyrosol and tyrosol at 280 nm; verbascoside at 330 nm (as chlorogenic-acid equivalent); and luteolin 7-O-glucoside and total flavonoids (as luteolin-7-O-glucoside equivalents) at 340 nm. All values are given as mg/kg oil or g/kg dw.

##### Tocopherol and Pigment Contents

For the analysis of *α-*, *γ-*, and *δ-*tocopherol, *β-*carotene, *α-*chlorophyll, lutein, and α-pheophytin, the oils were diluted in MTBE/methanol (90:10) to a final concentration of 20% *w/v* and the protocol of Cano et al. [22] was followed, with minor modifications.

An HPLC system was used, consisting of an LC-20AD pump, an SPD-M40 diode array (Shimadzu, Kyoto, Japan), an S 5300 (SYKAM, Eresing, Germany) sampler injector, and a 505 LC column oven (SSI, Mumbai, India). Separation was achieved on a C30 reverse-phase column (YMC-Pack YMC C30, 250 mm × 4.6 mm id, S-5 μm, YMC Co., Ltd., Kyoto, Japan) with a 1 mL/min flow rate. The mobile phase consisted of(A) MTBE/methanol (90:10), and (B) methanol/MTBE/water (81:14:4). The gradient elution protocol, with a total run period of 60 min, was as follows: 0 min, 100% B; 30 min, 50% B; 35 min, 0% B; 40 min, 0% B; 45 min, 100% B. A total of 20 μL of the oil dilution was injected in the system and identification was based on comparison of peak retention time and DAD spectra with available commercial standards at 294 nm (tocopherols), 450 nm (*β-*carotene and lutein), and 670 nm (*α-*chlorophyll and *α-*pheophytin) and results are expressed as mg/kg oil or g/kg dw (lutein and *α-*pheophytin are expressed as *β-*carotene and *α-*chlorophyll equivalents). To identify *α-*pheophytin, an acetone extract from fresh blanched spinach leaves treated with a few drops of concentrated HCl was analyzed under the same conditions.

##### Triterpenic Acid Content

The extraction of the triterpenic acids from the oil samples was performed according to the optimum conditions proposed by Olmo-Garcia et al. [23]. Briefly, 5 mL of methanol was added to 0.2 g of oil and shaked for 1 min. The mixture was left in an ultrasonic bath for 30 min and centrifuged at 2800× *g* for 6 min. These steps were repeated twice and the supernatants were combined, evaporated to dryness in a rotary evaporator (BÜCHI Labortechnik AG, Flawil, Switzerland) at 35 °C, and the residue was redissolved in 1 mL methanol.

Detection and quantification of the triterpenic acids, oleanolic (OA) and maslinic (MA), at 210 nm was carried out in the UHPLC system using the same column as for phenols and an isocratic elution system as follows: (A) water acidified with 0.2% H_3_PO_4_, and (B) methanol (92:8 *v/v*), with a flow rate of 0.8 mL/min [24]. The injection volume was 5 μL for the oils’ polar fraction and OL, and 2 μL for the OP (1:1 dilution) extract. Identification was based on the comparison of peak retention times and DAD spectra with standards, and results are expressed as mg/kg oil or g/kg dw.

### 2.10. Sensory-Based Attributes

#### 2.10.1. CIELab Color Evaluation

Color measurements were carried out using an aliquot (ca. 5 mL) of the translucent sample and a Hunter Miniscan XE Plus spectrophotometer (model 45/0 Large Area View (LAV), 2.54 cm aperture diameter, 10° standard observer, D65 illuminant; Hunter Laboratories, Reston, VA, USA). The rectangular coordinates L* (pure darkness 0/pure lightness 100), a* (green − /red +), and b* (blue − /yellow +) and the cylindrical ones, C* and h, were automatically calculated according to the CIEL*a*b* and CIEL*C*h color scales. The distance/difference/similarity (ΔΕ) between the color of C-VAO and each of the enriched oils was calculated using the formula ΔΕ = √ [(L_0_ − L_1_)^2^ + (a_0_ − a_1_)^2^ + (b_0_ − b_1_)^2^], where L_0_, a_0_, and b_0_ are the L*a*b* values of C-VAO; and L_0_, a_0_, and b_0_ are the L*a*b* values of the enriched C-VAOs. For each test sample, five replicate measurements were obtained. L*a*b* values were converted to the nearest standard color using open-access online software [25].

#### 2.10.2. GC-MS Analysis of Volatile Organic Compounds (VOCs)

VOC analysis was performed by Headspace Solid-Phase Microextraction (HS SPME) of the oils and analysis by gas chromatography–mass spectrometry (GC-MS) as described by Lioupi et al. [26]. Briefly, 2 mL of oil sample was put into a 15 mL glass vial, closed with a PTFE/silicone septum, and left in a PAL SHIMADZU autosampler unit (AOC 6000, CTC Analytics, Zwingen, Switzerland) at 55 ± 0.1 °C for 15 min, with agitation at 250 rpm. After their equilibrium, a PAL SPME fiber (DVB/PDMS/CAR WR/PDMS divinylbenzene/polymethylsiloxane/carbon wide-range/polymethylsiloxane Dual-Phase 80 μm, 50/30 thickness, Sigma-Aldrich, St. Louis, MO, USA) was introduced to the headspace for 50 min at 55 °C to absorb the volatiles. The fiber was pre- and post-conditioned for 10 min at 260 °C, according to the manufacturer’s instructions.

VOCs were analyzed using a Shimadzu GC-MSQP2020 (Shimadzu, Kyoto, Japan) instrument with a MEGA-5 MS capillary column (30 m × 0.25 mm, 0.25 µm; Legano, Italy). Injection was operated in split-mode (1:2) at 260 °C for 4 min, He was the carrier gas at a flow rate of 1.2 mL/min, the column was held for 2.5 min at 40 °C, then programmed to 230 °C at 10 °C/min, and held for 5 min. The mass detector was operated in the electron impact mode and scanned at m/z 35–450 amu range.

Compound identification was carried out by comparing the mass spectra of eluting compounds with those of the commercial library NIST17, considering the compounds with a score of ≥80% in similarity checks. The semi-quantitative results are expressed as the following: relative peak area (%) = (peak area of individual VOC/total peak area of identified VOCs) × 100.

### 2.11. Statistical and Principal Component Analysis (PCA)

All measurements per replicate sample were performed in triplicate and expressed as mean values ± standard deviation. Comparison of the mean values was carried out using SPSS Statistics v.28.0 software (IBM Corporation, Armonk, NY, USA), employing one-way analysis of variance, followed by Duncan’s range test at *p* < 0.05.

Principal component analysis (PCA) of the ATR-FTIR spectral data was performed using SIMCA 16.1 software (Umetrics, Umeå, Sweden). The input data matrix consisted of the average spectral intensities per replicate sample (n = 3) over the wavenumber range of 3600–2700 and 1800–650 cm^−1^ (10 observations × 1738 variables). The data were mean-centered, scaled, and the most important principal components (PCs) were evaluated according to the total variance explained (R_2_X(cum) ≥ 95%) and the Kaiser criterion (components with eigenvalues ≥ 1).

## 3. Results

### 3.1. Chemical Composition and Antioxidant Activity of Leaf and Pomace Materials

Compositional data about the total phenol content (TPC) and flavonoid content (TFL), the levels of the main individual phenols and triterpenic acids of the plant extracts, including antioxidant activity (DPPH^●^ and CUPRAC) are presented in Table 1.

AL was the richest in total phenols and antioxidant constituents but significantly poorer in total flavonoids. In particular, TPC was 1.38-fold higher in AL than in OL, in contrast to results reported by Jimenez et al. [8] (OL being 1.76-fold richer than AL). Its TPC was found to be much higher than that reported by other researchers for fresh avocado leaves [8,27] (29 vs. 3.7–19.3 mg GAE/g fresh leaves). In the case of OL and OP extracts, TPC values varied within a typical range for their botanical sources [9]. HR-MS and LC-DAD analyses of AL extracts verified the abundance of a phenolic acid (Table 1, Figure S1), putatively identified as chlorogenic acid [28] (*m/z* 353.0859 [M-H]^−^, *m/z* 191.0555 [M-H]^−^ for quinic acid residue). The same compound prevailed in the phenolic composition of hydroalcoholic extracts from AL cv. Hass [8]. In our study, the AL (cv. Fuerte) extract probably contained traces of procyanidin B1 [29] (*m/z* 245.0425 [M-H]^−^) and isoquercitrin (*m/z* 463.0872 [M-H]^−^) or another kaempferol di-glucoside (*m/z* 609.1478 [M-H]^−^). Quercetin-O-hexoside or other typical kaempferol derivatives that have been reported in the AL cv. Hass [8] were not detected.

HPLC-profiling data provided further insight into the potential of those plant materials as sources of VAO oxidation inhibitors. OL extracts were rich in well-known chain-breaking antioxidants such as oleuropein, luteolin-7-O glucoside, and verbascoside. Significant amounts of oleanolic and maslinic acids were also found. Although these metabolites are known as intracellular antioxidants [30], they are not expected to be strong radical scavengers under common in vitro assay or autoxidation conditions. Maslinic and oleanolic acids were also present in the olive pomace (OP) extract that was found to be richer in simple phenols rather than flavonoids. Thus, hydroxytyrosol, being almost 3-fold higher than tyrosol, was the only strong antioxidant identified in OP [31]. Beyond TPC, the HPLC-profiling data better explained the trends in AA of the studied samples after inter-comparison (Table 1). The activity was equivalent to 0.1–0.6 mol TE/kg (dw) of the same size as in recently reported data for agro-industrial by-products including avocado leaves [32]. Of note, the materials from olive fruit processing (dried leaves and pomace) performed better in CUPRAC than in the DPPH^•^ assay, also signifying that their antioxidant constituents may act through different mechanistic routes.

### 3.2. FT-IR Analysis of Enriched Oils

FT-IR analysis was carried out first as a non-destructive testing approach for the intact oil. The ATR-FTIR spectra of the virgin avocado oil (C-VAO, C-AO) contained all the characteristic bands of the fatty acid chains (methylene groups, cis–cis, or cis–trans double bonds), carbonyl and acyl ester bonds, as well as hydrogen-bonded molecular networks of freshly produced oils. These features coincided with the respective FTIR spectral bands of fresh EVOO [33] and are discussed in the following paragraphs. A high degree of unsaturation could be deduced from the spectral data in the region between 2850 and 3010 cm^−1^ based on earlier findings about the diagnostic importance of those bands [34,35]. Similarly, the ATR-FTIR spectra of the fortified avocado oils were visually identical to those of the reference oils. After normalization with the Standard Normal Variate (SNV), greater intensity variance was easily observed in the regions 650–780 cm^−1^, 1000–1250 cm^−1^, and 2850–2960 cm^−1^ (Figure S2). However, the patterns of variance according to the fortification agent and its addition level were visualized only after PCA, especially through the corresponding score and loading plots. To avoid overfitting, the data from the full spectral range were minimally processed (smoothed or averaged) before normalization and mean-centering. The dataset (10 oil samples × 1738 spectral points) resulted in four principal components (PCs) explaining 99% of the total variance. The major part (94.4%) could be described only by the first two PCs, indicating that the third and fourth PC carry systematic but minor differences among the spectra.

Visualization of the scattered t1/t2 scores over the first two PCs (Figure S3) revealed that most of the fortified oils were clouded close to zero. It is striking though that the PC1 and PC2 axes were formed mainly due to the differences between the base AO and those fortified with the lowest amount of leaf material, OL and AL (5% *w/w*), respectively. The p2 loading values (*p* > 0.05) signified that variance in three spectral regions—mainly at 2920–2960 cm^−1^, along with those at 1730–1740 cm^−1^, and less importantly at around 3450 cm^−1^—accounted for the scattering across PC2. On the other hand, distinct patterns of variance at around 720, 1140–1160, along with 2850–2860, 2910, and 1740–1750 cm^−1^ were revealed from the lowest/highest p1 loadings. These results imply that methylene and carbonyl bond-stretching vibrations are the major contributors to the exposed pattern. The variance at 3450 cm^−1^ also indicates a role of N-H bonds (as in amide groups), while that at 2910 cm^−1^ could arise from alkane residues attached to other hetero-atoms [35]. One can speculate that AO fortification with the lowest dose of leaf material provoked changes in the content and configuration of unsaturated bonds of lipid and/or protein molecules that were not replicated with higher doses. Exploration of the lower PCs in the modeled spectral data did not add any further information regarding the possible physicochemical changes due to fortification with OP. Further analyses were thus carried out to enrich our understanding of those phenomena.

### 3.3. Quality Parameters

Typical quality parameters including the free fatty acid content, peroxide values, and extinction coefficient values (K_232_, K_270_) of the oils under study are shown in Table 2. Based solely on the low FFA content (less than 0.5% oleic acid), the laboratory-produced AO that was used as the base oil for enrichment experiments could be characterized as “extra virgin” oil. Existing reports on the quality characteristics of commercial virgin AOs sold in the USA or Chilean markets showed that FFA content may vary widely, even exceeding 2.50% [5,36]. Considering the literature peroxide values for laboratory-extracted virgin AOs (1.4 to 12.74 meq O_2_/kg), our base oil was fresh, indicating the high quality of fruits and/or careful handling during processing and storage. The same was evidenced for C-AO, even though it presented much higher FFA and PV values than the lab-extracted one. It is stressed that VAO should contain no more than 1% FFA and 15 meq O_2_/kg according to trade standard specifications [37]. The freshness and high quality of C-VAO and C-AO were also verified after estimation of the K_232_ and K_270_ values (Table 2), providing information about conjugated dienes and trienes formed upon lipid oxidation, respectively. These values were quite low compared either to the K_232_ ones reported by Ramírez-Anaya et al. [38] (1.8–2.8) or the K_270_ values reported by Elen-Martinez et al. [39] for just-processed, centrifuge-extracted oils (0.37–0.45).

All of the fortified AO samples presented relatively low FFAs (less than 0.5% oleic acid) and PVs (less than 4.0 meqO_2_/kg oil), signifying that they retained freshness. Nevertheless, PVs varied more widely in OL-VAOs (1.6 to 3.9) than in AL-VAOs (2.7 to 2.9) or in OP-VAOs (1.6 to 2.8), and their K_232_ and K_270_ values varied within the ranges reported in the literature for fresh VAOs [3,4]. AL20 was the only exception. Based on the results from UV-based indices of oxidation, it seems that when avocado leaf powder was added at the highest level examined in this study (20% *w/v*), it promoted lipid autoxidation, an observation not verified by PV determination. This finding must be interpreted with caution. Other UV-absorbing constituents deriving from the fortification agent (e.g., chlorogenic acids) could interfere with the signals of primary oxidation products, altering the specificity and, thus, limiting the usefulness of K_232_ and/or K_270_ as oil “freshness” indicators. This is probably the reason why a linear dose-dependent increase was observed upon enrichment of VAO with plant agents. This particular concept has been discussed again in the literature, highlighting potential quality control pitfalls [40].

### 3.4. Oxidative Stability

The induction period (IP), the predicted induction period (p-IP), and the protection factor (PF) values of each oil tested under the accelerated autoxidation conditions of the Rancimat test are shown in Table 3. Both C-VAO and C-AO presented the lowest oxidative stability at 20 °C (IP C-VAO: 19.42 h; IP V-AO: 20.28 h) among the tested oils. Their p-IP value was estimated to be almost 14 months, 4 months earlier than that proposed by Woolf et al. [4]. On the other hand, the control olive oil sample, which was of the extra grade (EVOO) in terms of quality parameters (Table S1), was the most resistant to oxidation (p-IP = 24.89 months). These findings add to the scope of the present study. While the oxidative stability of VAO increased upon fortification with different plant materials in a dose-dependent manner, the improvement was almost negligible in the case of AL. At the highest addition level, the p-IP value of AL-VAO reached 17.26 months. This finding is not aligned with the strong response of AL extracts using in vitro AA assays (Table 1). On the other hand, OP exhibited superior performance regarding the protection effect, followed by OL. Specifically, the p-IP values of OP-enriched VAOs extended from 14 (C-VAO) up to 22 months (OP20), providing a significant contribution to the shelf-life of the product.

### 3.5. Antioxidant Activity

**Figure 1:**
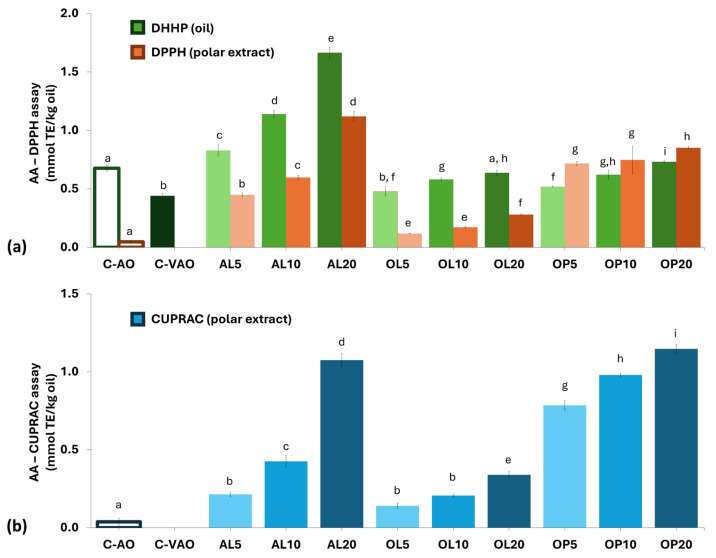
Antioxidant activity (AA) of oil samples, using both DPPH^●^ (**a**) and CUPRAC (**b**) assays. C-AO = avocado oil; C-VAO = freshly extracted virgin avocado oil; AL5-AL10-AL20, OL5-OL10-OL20, and OP5-OP10-OP20 represent C-VAO enriched with avocado leaves, olive leaves, and olive pomace, respectively, at 5, 10, and 20% (*w/w*) employing ultrasound-assisted maceration. Results expressed as mean value ± standard deviation (*n* = 3). Darker colors per used plant material indicate higher enrichment levels. Different letters above the bars indicate significant differences (*p* < 0.05) within the whole sample population, per assay, employing Duncan’s test.

The DPPH^•^ scavenging or Cu-reducing activities of the oil polar extracts and the DPPH^•^ scavenging activity of the whole oil, expressed in terms of Trolox equivalents (on an mM basis), are shown in Figure 1. The whole oil was examined to include the contribution of lipophilic antioxidants (e.g., tocopherols) except for the polar ones. C-VAO presented the lowest values of AA, calculated either in intact oil (0.44 mmol TE/kg oil) or in polar extract form. A similar trend was observed in the case of C-AO, verifying that the particular oils are devoid of polar antioxidants.

The values indicated that C-VAO is not expected to contain efficient polar antioxidants, only lipophilic ones. A similar observation was made for C-AO. AA increased upon fortification with the plant materials in a dose-dependent manner, reaching up to 1.66 mmol TE/kg in the case of AL20 when tested in its intact form, surpassing that of C-EVOO (1.52) used for comparison. A dose-dependent trend in activity was also observed in the case of the isolated polar fraction. In that case, the AL20 reached the same level of activity as that of C-EVOO (1.12 vs. 1.14 mmol TE/kg oil) in terms of the DPPH assay but this was significantly less active when CUPRAC was applied (1.07 vs. 2.34 mmol TE/kg oil). These results do not coincide with the poor performance of AL-VAOs under accelerated autoxidation conditions. However, it may indicate that polar antioxidants are transferred to VAO upon maceration of the plant materials.

### 3.6. Chemical Composition

#### 3.6.1. Fatty Acid Content and Oxidative Susceptibility

To better understand the findings so far, we examined the chemical composition of the oils, starting from the % content of fatty acids (Table S2). Our results verified that the avocado oils were rich in monounsaturated fatty acids (MUFAs), as also the EVOO, with a composition in accordance with quality specifications [40] and previous research [6,41]. Regarding the fortified avocado oils, the FA composition was also similar to that of the base AO (C-VAO). The relevant data in the literature indicate that other fortification process parameters may be significant. For example, Baccouri et al. [42] found that maceration of olive oils with olive leaf material did not affect their fatty acid composition, while Arfaoui et al. [43] reported a significant increase in the oleic/linoleic acid content ratio upon ultrasound-assisted extraction. The increase in oleic acid was attributed to extraction from the leaf, whereas the relatively small decrease in linolenic acid was due to ultrasound-mediated oxidation. The latter may occur due to thermal effects and/or the formation of free radicals in edible oils [44]. In our study, fortification did not induce any appreciable effect on the % MUFA content (Table 4). On the other hand, the ratios SFA/PUFA and MUFA/PUFA became slightly higher but only at the lowest addition level (AL5, OL5, and OP5). As a result, the oxidation susceptibility (OS) index, which relies heavily on the content of highly oxidizable FAs, followed a similar pattern of changes.

These observations signify that during the ultrasound-assisted maceration at the lowest addition level, PUFAs are not efficiently protected against the oxidation that is expected to be promoted by the applied technique. It is interesting to note that a similar pattern of variance among the studied oils was also evidenced through the PCA-based analysis of the ATR-FTIR spectral data. As these changes may take place at room temperature, we cannot explain the performance of the fortified VAOs under thermally induced oxidation conditions, such as in the Rancimat test.

#### 3.6.2. Phenolic Compound and Triterpenic Acid Contents

**Figure 2:**
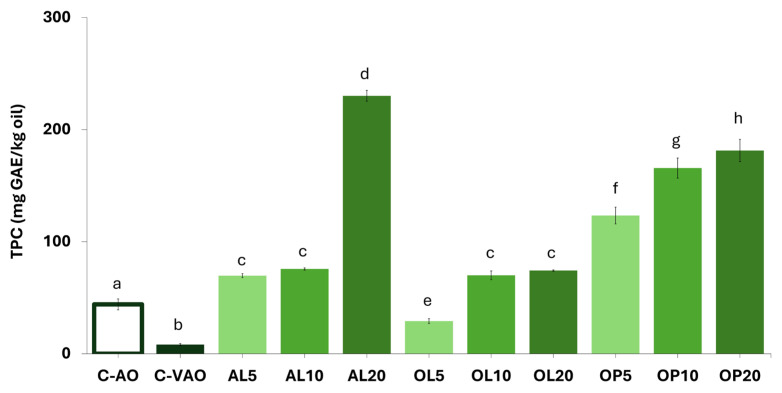
Total phenolic content (TPC) of oil samples. C-AO = avocado oil; C-VAO = freshly extracted virgin avocado oil; AL5-AL10-AL20, OL5-OL10-OL20, and OP5-OP10-OP20 represent C-VAO enriched with avocado leaves, olive leaves, and olive pomace, respectively, at 5, 10, and 20% (*w/w*) employing ultrasound-assisted maceration. Results expressed as mean value ± standard deviation (*n* = 3). Darker colors per used plant material indicate higher enrichment levels. Different letters above the bars indicate significant differences (*p* < 0.05) within the whole sample population employing Duncan’s test.

Figure 2 illustrates the variance in the TPC values of the studied oils. VAO was almost devoid of phenols (8.2 mg GAE/kg oil), as estimated according to the Folin–Ciocalteu assay. C-AO was richer but both of them were poor compared to C-EVOO. The base oil was significantly enriched with phenols upon fortification with any of the three types of agents. In particular, OP performed better than OL or AL as an enrichment agent at the low addition levels used (5–10%, *w/v*). This result drew our attention given the compositional data of the plant material presented in Table 1. Moreover, the fortification experiments showed that enrichment with AL- or OL-derived phenols was not dose-dependent. Among all, AL20 was by far the richest in total phenols (230 mg GAE/kg oil), closer to the content found for C-EVOO (349 mg GAE/kg oil). An insight into the phenolic composition of the studied oils through HPLC-DAD analyses (Figure S4) revealed that chlorogenic acid, oleuropein, and hydroxytyrosol/tyrosol—as the major phenolic constituents of AL, OL, and OP, respectively—were identified in all samples of AL-, OL-, and OP-VAOs. Their contents increased with the addition level, as opposed to the respective Folin–Ciocalteu responses. Thus, AL- and OL-VAOs with less than 25–30 mg of total major phenols /kg oil (e.g., AL5, AL10, and OL5) were considered poor in antioxidants and more prone to oxidation phenomena.

**Figure 3:**
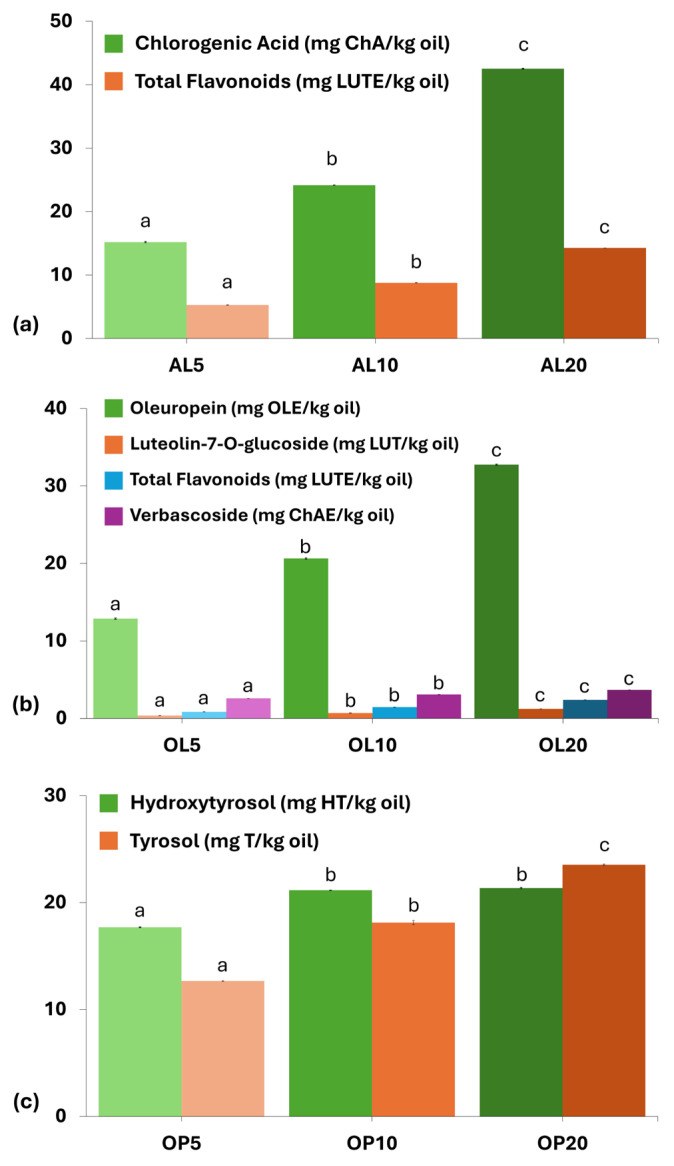
Phenolic content of oils enriched with (**a**) avocado leaves (AL), (**b**) olive leaves (OL), and (**c**) olive pomace (OP). AL5-AL10-AL20, OL5-OL10-OL20, and OP5-OP10-OP20 represent freshly extracted virgin avocado oil enriched with avocado leaves, olive leaves, and olive pomace, respectively, at 5, 10, and 20% (*w/w*) employing ultrasound-assisted maceration. Results expressed as mean value ± standard deviation (*n* = 3). Darker colors per used plant material indicate higher enrichment levels. Different letters above the bars indicate significant differences (*p* < 0.05) within samples macerated with the same plant material per compound, employing Duncan’s test.

Using liquid chromatography as a more selective analytical technique, it was evidenced that polar antioxidants present in the corresponding material were transferred to C-VAO (Figure 3). A similar transfer of oleuropein and other polar biophenols from OL to VOO was previously reported, using dynamic or static approaches employing ultrasound [45,46].

The transfer was small when considering the levels measured in the materials after examination of their extracts (Table 1). The level achieved for oleuropein was almost similar to that (20 vs. 14.4 mg/mL) reported by Japón-Luján et al. [45] using a dynamic approach comprising an ultrasonic probe at the same level of OL addition with 20 min of extraction. Similar values were observed for luteolin glucoside and verbascoside. However, the level of oleuropein was inferior to that achieved by the static bath-type ultrasound of Achat et al. [46], who, within 45 min of processing and 15% of OL addition, managed to transfer 111 mg OLE/kg oil. Even the addition of OL at 20% resulted in no more than 34 mg OLE/kg oil. However, these values are well below those of a high-quality EVOO, such as the one included in the present study (total hydroxytyrosol: 152 mg/kg; total tyrosol: 372 mg/kg). Similar observations were made upon AL addition, although the maximum level of chlorogenic acid found at AL20 was higher (almost 42 mg/kg oil) than that of oleuropein in OL20. The level of hydroxytyrosol was even lower in OP20. The enrichment in tyrosol, though of interest in terms of bioactivity [47], is not expected to contribute to the oxidative stability of the oil [31].

**Figure 4:**
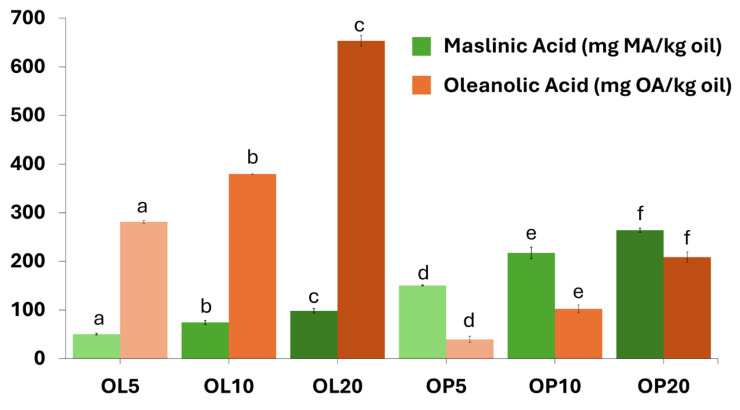
Triterpenic acid content of oils enriched with olive leaves and pomace. MA = maslinic acid; OA = oleanolic acid; OL5-OL10-OL20 and OP5-OP10-OP20 represent freshly extracted virgin avocado oil enriched with olive leaves and olive pomace, respectively, at 5, 10, and 20% (*w/w*) employing ultrasound-assisted maceration. Results expressed as mean value ± standard deviation (*n* = 3). Darker colors per used plant material indicate higher enrichment levels. Different letters above the bars indicate significant differences (*p* < 0.05) within samples macerated with OL and OP per compound, employing Duncan’s test.

Contrary to the findings on polar phenols, the enrichment with triterpenic acids in the cases of OL and OP was significant. The levels were several-fold higher considering the lipophilic nature of the compounds and the corresponding levels in the plant materials. As evident, depending on the material used (OL or OP), the end product could be either rich in maslinic or oleanolic acid (Figure 4).

Thus, with 20% OL, oleanolic acid reached up to 653 mg/kg oil (almost 100 mg/kg oil in maslinic acid), whereas with 20% OP, maslinic acid reached 264 mg/kg oil (almost 200 mg/kg oil in oleanolic acid). Their presence in the oils—except for enhancing the content of bioactives [48]—may contribute to the oxidative stability, as reported by Orozco et al. [49] who found that they become depleted in enriched oils upon repeated frying. The same authors commented that in oils enriched with OP extracts, maslinic acid may be present in a conjugated form as well that is liberated during the first heating cycles, resulting in an apparent increase in its levels and, thus, contributing further to oil protection.

#### 3.6.3. Tocopherol Content

Tocopherols are the lipophilic antioxidants present in plant edible oils. According to Psomiadou et al. [50] on the monitoring of the quality of VOO, their contribution is positive to the OSI value, though ~2.5-fold less than polar phenols (determined with Folin–Ciocalteu assay). In contrast to the observed effect on FA composition, only AL increased significantly in the total tocopherol content of the base AO. Thus, AL10 and AL20 were enriched 2–3-fold, reaching a content of more than 400 mg/kg oil, much higher than those of commercial AO or EVOO (244.61 mg/kg oil) samples. Three tocopherol analogs (*α-*, *γ-*, and *δ-*) were identified and quantified (Table 5) in the avocado oil samples, with *α-*Τ being the major one, as expected [1,51]. Both *γ-*Τ and *δ-*Τ represented 35–45% of the total tocopherol content of pure AOs, substantially higher than in C-EVOO [52]. This point needs some attention because *γ-*Τ and especially *δ-*Τ are known to be rather negligible in avocado fruit oils [4]. Recently published data about the composition of AOs from unpeeled avocado fruits signify that other plant tissues might be responsible for elevated levels of those tocopherols [53].

Fortification with avocado leaf (but not with olive leaf or olive pulp) enriched the base AO mainly in *α-*T. This is an important finding considering both the antioxidant and the vitamin activity of the respective analog. The low enrichment of AO in the case of OL (up to 1.2-fold at 20%) was expected when taking into account some limited reports in the literature where no more than 1.8-fold increase has been reported [42,46,54]. The lack of any influence upon the addition of OP is probably related to the fact that a low content is expected in such material (~157 mg/kg) as found by Gracia et al. [55], despite the use of the selective supercritical extraction after optimization. In addition, the high drying temperature (140 °C) used could have promoted vitamin E losses in the plant material.

#### 3.6.4. Carotenoid and Chlorophyll Contents and Profiles

**Figure 5:**
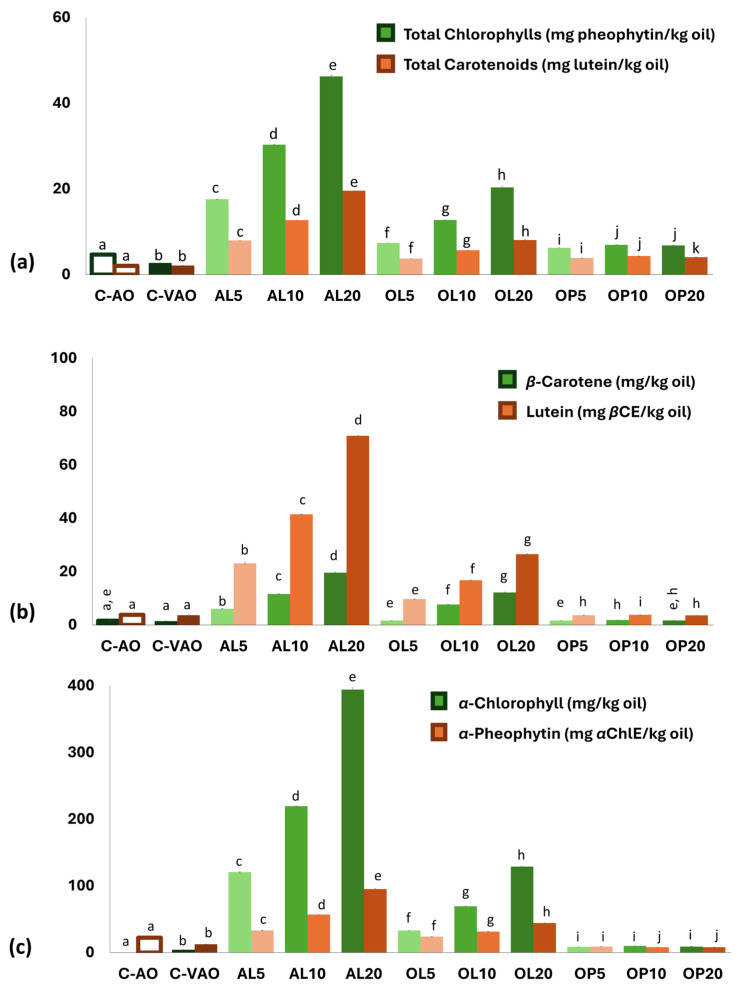
Pigments of oils: (**a**) total chlorophyll and carotenoid contents assessed by UV–Vis spectroscopy, and (**b**,**c**) levels of selected pigments determined via HPLC-DAD. βCE = *β-*carotene equivalents; αChlE = *α*-chlorophyll equivalents; C-AO = avocado oil; C-VAO = freshly extracted virgin avocado oil; AL5-AL10-AL20, OL5-OL10-OL20, and OP5-OP10-OP20 represent C-VAO enriched with avocado leaves, olive leaves, and olive pomace, respectively, at 5, 10, and 20% (*w/w*) employing ultrasound-assisted maceration. Results expressed as mean value ± standard deviation (*n* = 3). Darker colors per used sample material indicate higher enrichment levels. Different letters above the bars indicate significant differences (*p* < 0.05) within the whole sample population per compound, employing Duncan’s test.

Carotenoids, chlorophyll, and pheophytin determination were carried out as they are reported to to contribute to the oxidative stability of edible oils. More specifically, according to Psomiadou et al. [50], who worked on the monitoring of the quality of VOO, it was revealed via modeling a positive contribution of the total chlorophyll content to the determined OSI value, which was 1.8-fold higher than that of the TPP content. It should be stated that their sample population included oils rich in chlorophylls, similar to those of the present study (Figure 5). Carotenoids such as *β-*carotene, although when added alone in bulk oils may be inactive or even show pro-oxidant activity, in the presence of *α-* and *γ-*tocopherol they may act as efficient antioxidants due to synergism [56]. Examination of the determined values indicated that the content of the two pure AOs in total carotenoids ranged within values reported in the literature for cold-pressed avocado oil [4,53] (0.9–3.5 mg/kg oil). AL and OL addition significantly enriched the carotenoid content of AO, in a dose-dependent way, up to 20 and 8 mg/kg, respectively. It is of note that, apart from the content, the profile of carotenoids changed as the oils became gradually richer in xanthophylls (expressed as lutein) than in carotenes (expressed as *β-*carotene). Moreover, AL-fortified oils were 2.5–3-fold richer than OL ones in both types of constituents, signifying the superior contribution of avocado leaves.

Regarding chlorophylls and related compounds, the HPLC analysis verified that C-VAO was a fresh, high-quality oil that retained *α-*chlorophyll. The total chlorophyll content was rather low if we consider that Green and Wang [36] reported a range in the literature of 1–69.8 mg/kg and, in their work, after analyzing several commercial samples, a range of 6.62 to 98.8 mg/kg, which was explained by the authors as possibly due to the inclusion of the skin upon processing. Despite the fact that at the highest level of the plant material used, a dark green coloration was obtained—as in AL20—and the corresponding value using two different analytical protocols was within the range reported above for commercial AO samples. Furthermore, it should be stressed that even commercial EVOOs from cv. Koroneiki can be very rich in total chlorophylls (64.1 mg /kg oil) as it has been reported in the literature for Greek oils of cv. Koroneiki [57]. Focusing on the individual pigments for the β-carotene, α-chlorophyll, lutein, and α-pheophytin composition in oil samples, it was evident that from 1.46, 4.24, 3.69, and 12.54 mg/kg in the control sample (C-VAO), the levels reached up to 29.06, 394.19, 70.96, and 95.37 mg/kg (AL20), respectively. Considering that light exposure can be avoided through packaging, it is important to stress that various chemopreventive activities against different diseases, including cancer, have been reported for chlorophylls [58]. The β-carotene and lutein also provide additional nutritional value to the oils due to their pro-vitamin-A activity [59].

### 3.7. Contribution of Chemical Composition to the OSI and AA of the Oils

Taking into account the information obtained so far, Pearson’s correlation analysis was employed to see whether statistically significant correlations could be found between the IP and AA values or chemical constituents present in the examined samples (base and fortified AOs). The analysis showed a significant correlation between the IP values recorded for the samples and the TPC (*r* = 0.796, *p* = 0.002) or AA (DPPH^●^: *r* = 0.676, *p* = 0.016; CUPRAC: *r* = 0.841, *p* = 0.001). In the cases where whole oils were examined with DPPH^●^, a significant correlation of AA was evidenced with the content in the lipophilic α-tocopherol (*r* = 0.881, *p* = 0.000) and, to a lower extent, with the total chlorophyll (*r* = 0.608, *p* = 0.036) and total carotenoid (*r* = 0.591, *p* = 0.043) levels. Considering the findings for the IP, it is evident that polar compounds including polar phenols contribute the most. The latter is partially in agreement with the findings of Psomiadou et al. [50] that have already been discussed in previous paragraphs, who highlighted the positive contribution to EVOO (cv. Koroneiki) of total polar phenols, determined with the Folin–Ciocalteu assay, as no correlation was found for α-tocopherol or total chlorophyll content determined spectroscopically.

### 3.8. Color and VOC Profile

Beyond oxidative stability, sensory attributes, such as color, odor, and flavor, as well as biological and microbial safety issues of the enriched oils need to be evaluated to be acceptable as novel products, issues that are often neglected by researchers [1]. In the present study, the color and volatile organic compound (VOC) profile of the oils were evaluated due to the limited volume of samples.

#### 3.8.1. Color

**Figure 6:**
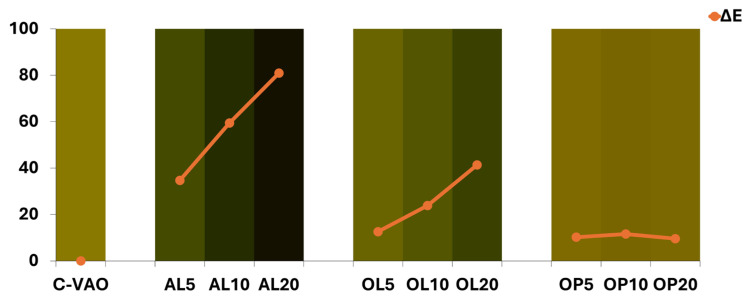
Visual appearance of the studied virgin avocado oils. Orange lines represent the color similarity (ΔΕ, mean values, *n* = 5) of the enriched oils with the control. C-AO = avocado oil; C-VAO = freshly extracted virgin avocado oil; AL5-AL10-AL20, OL5-OL10-OL20, and OP5-OP10-OP20 represent C-VAO enriched with avocado leaves, olive leaves, and olive pomace, respectively, at 5, 10, and 20% (*w/w*) employing ultrasound-assisted maceration.

The CIELab color parameters, including Chroma and hue, are provided in Table S3. The freshly extracted C-VA appeared more green and yellow than C-VAO and C-EVOO (Table S1), considering the a* and b* values. In Figure 6, the visual perception of the studied oils through appropriate conversion of the CIELab color parameters is depicted, including the ΔE values derived from the comparison of C-VAO to the corresponding fortified AOs. Pictures of samples are provided in Figure S5. Above all, the addition of the leaf material (avocado or olive origin) had a dose-dependent negative effect on L* and C* values, signifying that the gradual darkening brought about significant loss of oil brightness and color saturation. This effect was stronger in the case of AL- vs. OL-fortified AOs but it was not evidenced in the case of OP-fortified oils. In the latter case, the a*/b* ratio and hue (ho) values slightly increased, causing a subtle shift toward a deeper yellow–orange color. Still, in this case, the ∆E values were much larger than the threshold of three, meaning that the color differences can be visually perceived for these kinds of samples [60]. Clearly, inspecting the visual conversion, samples fortified with AL at 10% and 20% were too dark to be acceptable, although the green pigment levels were within the range of commercial samples. Overall, our results indicate that the color of the enriched AOs could be a critical factor to be evaluated in future consumer acceptance studies of these products as “virgin” and minimally processed products.

#### 3.8.2. VOC Profile

The volatile organic compound profile of C-VAO (Table S4) was dominated by aldehydes (43.19%), followed by acids (22.62%) and hydrocarbons (19.05%), whereas esters, alcohols, and ketones had little contribution. The major constituent was 2-hexenal (20.79%), contributing to herbal and sweet notes, followed by (E)-caryophyllene (3-fold less), also with sweet notes [61].

Upon fortification with avocado leaf material, the VOC profile changed dramatically. In particular, estragole followed by methyl-eugenol became the most abundant constituents (~50% and 13–20%, respectively), followed by hydrocarbons (~20%). Estragole, with an anise-like odor, and methyl-eugenol, with a spicy earthy odor and a bitter burning taste [61,62], are strong biomarkers of AL. The concentration of these compounds in fortified edible oils should be carefully monitored given their genotoxic/hepatotoxic activity in rodents [63]. Currently, no definitive study has been performed in the absence of hepatotoxicity using the oral route of exposure to estragole. While it is estimated that the average food intake is 0.5–5 mg estragole per day, the official recommendations refer to occasional uses of naturally rich herbal products as foods rather than supplements [64,65].

On the other hand, fortification with dried OL to the highest level (20% *w/w*) increased acids over aldehydes (50 vs. 20%), with acetic acid expected to impart an undesirable (irritant) vinegar perception [66], being prominent, while it was not detected at lower addition levels. At lower addition levels (5 and 10% *w/w*), aldehydes were prominent (40.2 and 33.2%, respectively), with 2-hexenal being the dominant compound in OL5 (19.7%) and hexanal adding green apple notes [67] in OL10 (24.2%).

Enrichment with OP resulted in the prevalence of acids (31–43%), ketones (13–23%), hydrocarbons (15–22%), and aldehydes (11–23%). Some major odorants were nonanoic and acetic acids (7–17% and 8–14%) and 2-hexenal (5–8%), which may impart milk [66], unpleasant vinegar, and typical fresh green odors, respectively [67]. Considering the fact that sensory perception is multiparametric and the above results are qualitative, deeper studies are required to objectively evaluate the aroma of the flavored products. Nevertheless, optimum handling conditions to reduce off-odors prior to use as a flavoring agent maybe necessary [68,69].

## 4. Conclusions

The present findings indicate that ultrasound-assisted maceration of VAO with powders of avocado leaves (AL) or olive processing by-products (OL and OP) may increase its content in various bioactives, mainly of lipophilic nature, in a dose-dependent manner. Regardless of the fortification agent used, the oxidative stability and antioxidant potential improved, while standard quality parameters remained within the typical ranges for VAOs. Although the oxidative stability correlated significantly with the total polar phenol content, further insight is required to better understand such findings considering that the HPLC analysis indicated low enrichment in strong phenolic antioxidants. Color alteration, especially in the case of AL-VAOs, may not necessarily be an issue for acceptability considering the literature findings for commercially available samples. The analysis of the VOCs indicated some possible safety and sensory issues. The presence of estragole and methyl-eugenol in the AL-VAOs or odorants in OP-VAOs and OL-VAOs, with potential unpleasant contributions, must not be overlooked in future studies. In the same direction, further research on the handling practices of raw materials to reduce off-flavors may be beneficial for oil manufacturers.

## Figures and Tables

**Table 1:** Compositional and antioxidant activity data for avocado leaves, olive leaves, and olive pomace material used in this study.

	AL	OL	OP
Colorimetry			
TPC (g GAE/kg dw)	73.78 ± 5.11	53.24 ± 1.33	16.28 ± 3.62
TFC (g QUE/kg dw)	3.92 ± 0.21	10.17 ± 0.33	0.61 ± 0.01
HPLC analysis			
Oleuropein (g/kg dw)	ND	53.18 ± 0.26	ND
Luteolin-7-O-glucoside (g/kg dw)	ND	3.33 ± 0.00	ND
Total flavonoids (g LUTE/kg dw)	5.01 ± 0.00	7.45 ± 0.71	ND
Chlorogenic acid (g/kg dw)	13.56 ± 0.04	ND	ND
Verbascoside (g CHAE/kg dw)	ND	3.86 ± 0.05	ND
Hydroxytyrosol (g/kg dw)	ND	ND	3.21 ± 0.03
Tyrosol (g/kg dw)	ND	ND	1.15 ± 0.00
Maslinic acid (g/kg dw)	ND	4.54 ± 0.05	6.05 ± 0.01
Oleanolic acid (g/kg dw)	ND	17.49 ± 0.28	1.80 ± 0.03
Antioxidant activity			
AA (DPPH, mol TE/kg dw)	0.56 ± 0.01	0.25 ± 0.01	0.09 ± 0.01
AA (CUPRAC, mol TE/kg dw)	0.55 ± 0.01	0.46 ± 0.06	0.17 ± 0.01

AL = avocado leaves; OL = olive leaves; OP = olive pomace; TPC = total phenolic content; GAE = gallic-acid equivalents; TFC = total flavonoid content; QUE = quercetin equivalents; AA = antioxidant activity; TE = Trolox equivalents; LUTE = luteolin-7-O-glucoside equivalents; ND = not detected. Results are expressed as mean value ± standard deviation (*n* = 3).

**Table 2:** Quality parameters of the studied control and enriched oils.

Sample	Free Fatty Acid (FFA) Content (% Oleic Acid)	Peroxide Value (PV) (meq O_2_/kg Oil)	K_232_	K_270_
C-AO	0.93 ± 0.03 a	5.86 ± 0.47 a	1.72 ± 0.02 a	0.18 ± 0.01 a
C-VAO	0.22 ± 0.00 b	1.74 ± 0.26 b	2.23 ± 0.00 b	0.24 ± 0.00 b
AL5	0.30 ± 0.03 c	2.93 ± 0.05 c,d	1.94 ± 0.06 c	0.30 ± 0.06 c
AL10	0.37 ± 0.03 d,e	2.44 ± 0.48 c,d	2.16 ± 0.02 d	0.42 ± 0.01 d
AL20	0.43 ± 0.03 e,f	2.73 ± 0.28 c,d	2.72 ± 0.01 e	0.76 ± 0.01 e
OL5	0.35 ± 0.03 c,d	2.45 ± 0.49 c,d	1.61 ± 0.00 f	0.12 ± 0.00 f
OL10	0.35 ± 0.03 c,d	1.58 ± 0.22 b	1.87 ± 0.02 g	0.19 ± 0.00 a
OL20	0.35 ± 0.03 c,d	3.94 ± 0.48 e	2.08 ± 0.03 h	0.28 ± 0.02 b,c
OP5	0.37 ± 0.03 d,e	2.13 ± 0.28 b,d	1.85 ± 0.00 g	0.24 ± 0.00 b
OP10	0.43 ± 0.03 e,f	1.64 ± 0.28 b	1.86 ± 0.04 g	0.29 ± 0.03 b
OP20	0.48 ± 0.03 f	2.81 ± 0.28 c,d	2.00 ± 0.04 i	0.35 ± 0.04 g

C-AO = avocado oil; C-VAO = freshly extracted virgin avocado oil; AL5-AL10-AL20, OL5-OL10-OL20, and OP5-OP10-OP20 represent C-VAO enriched with avocado leaves, olive leaves, and olive pomace, respectively, at 5, 10, and 20% (*w/w*). Results are expressed as mean value ± standard deviation (*n* = 3). Different lowercase letters in the same column indicate significant differences (*p* < 0.05) within the whole sample population employing Duncan’s test.

**Table 3:** Oxidative stability of oil samples using the Rancimat apparatus (110 °C, 20 L/h).

Sample	Induction Period, IP (h)	Predicted Induction Period, p-IP (months, 20 °C)	Protection Factor, PF
C-AO	20.28 ± 0.27 a	14.42 ± 0.19 a	-
C-VAO	19.42 ± 0.18 b	13.81 ± 0.13 b	-
AL5	22.17 ± 0.57 c	15.77 ± 0.40 c	1.14 ± 0.03 a
AL10	22.68 ± 0.40 c	16.13 ± 0.28 c	1.17 ± 0.02 a
AL20	24.27 ± 0.36 d	17.26 ± 0.26 d	1.25 ± 0.02 b
OL5	22.29 ± 0.07 c	15.85 ± 0.05 c	1.15 ± 0.00 a
OL10	25.40 ± 0.18 e	18.06 ± 0.13 e	1.31 ± 0.01 c
OL20	29.11 ± 0.21 f	20.70 ± 0.15 f	1.50 ± 0.01 d
OP5	26.30 ± 0.14 g	18.70 ± 0.10 g	1.35 ± 0.01 e
OP10	29.44 ± 0.64 f	20.93 ± 0.45 f	1.52 ± 0.03 d
OP20	31.35 ± 0.58 h	22.29 ± 0.41 h	1.61 ± 0.03 f

C-AO = avocado oil; C-VAO = freshly extracted virgin avocado oil; AL5-AL10-AL20, OL5-OL10-OL20, and OP5-OP10-OP20 represent C-VAO enriched with avocado leaves, olive leaves, and olive pomace, respectively, at 5, 10, and 20% (*w/w*). Results are expressed as mean value ± standard deviation (*n* = 3). Different lowercase letters in the same column indicate significant differences (*p* < 0.05) within the whole sample population employing Duncan’s test.

**Table 4:** Total saturated, mono-, and polyunsaturated fatty acid contents and oxidation stability index.

	C-AO	C-VAO	AL5	AL10	AL20	OL5	OL10	OL20	OP5	OP10	OP20
SFA	16.73	15.86	16.14	15.80	16.08	16.21	15.96	15.94	16.10	15.81	15.85
MUFA	73.53	75.32	75.64	75.82	75.06	75.85	75.51	75.13	76.04	75.92	75.43
PUFA	9.74	8.83	8.22	8.38	8.85	7.94	8.54	8.93	7.86	8.27	8.72
TUFA	83.27	84.15	83.86	84.20	83.92	83.79	84.04	84.06	83.90	84.19	84.15
PUFA/SFA	0.58	0.56	0.51	0.53	0.55	0.49	0.54	0.56	0.49	0.52	0.55
TUFA/SFA	4.98	5.31	5.20	5.33	5.22	5.17	5.27	5.27	5.21	5.33	5.31
OS	537.2	490.2	458.2	467.5	492.4	442.8	474.9	496.1	442.0	460.9	484.0

C-AO = avocado oil; C-VAO = freshly extracted virgin avocado oil; AL5-AL10-AL20, OL5-OL10-OL20, and OP5-OP10-OP20 represent C-VAO enriched with avocado leaves, olive leaves, and olive pomace, respectively, at 5, 10, and 20% (*w/w*). SFA = saturated fatty acids; MUFA = monounsaturated fatty acids; PUFA = polyunsaturated fatty acids; TUFA = total unsaturated fatty acids; OS = oxidation stability index. Results calculated by the mean values of the peak ratios (%) of individual fatty acids (*n* = 3).

**Table 5:** Tocopherol composition of avocado oil samples (mg/kg oil) expressed as α-Tocopherol equivalents.

Sample	*α-*Tocopherol	*γ-*Tocopherol	*δ-*Tocopherol	Total Tocopherols
C-AO	208.08 ± 10.18 a	74.14 ± 1.91 a	66.19 ± 3.68 a	348.41 ± 2.42 a
C-VAO	125.42 ± 2.21 b	34.25 ± 0.64 b	36.74 ± 0.24 b,c,d	196.41 ± 2.85 b
AL5	207.27 ± 2.00 a	39.01 ± 0.21 c	37.88 ± 0.78 c,d	284.17 ± 8.68 c
AL10	306.92 ± 1.22 c	48.50 ± 4.72 d	40.57 ± 5.33 d	395.99 ± 14.23 d
AL20	473.73 ± 12.94 d	53.43 ± 1.22 e	50.42 ± 1.81 e	577.59 ± 1.94 e
OL5	125.79 ± 0.65 b	33.07 ± 0.70 b	37.44 ± 0.75 c,d	196.30 ± 3.99 b
OL10	147.31 ± 0.94 e	36.68 ± 1.18 c	39.97 ± 3.87 d	223.97 ± 1.02 f
OL20	154.05 ± 0.59 e	33.27 ± 0.66 b	38.54 ± 1.02 c,d	225.86 ± 3.79 f
OP5	132.19 ± 2.62 b,f	33.21 ± 0.79 b	35.27 ± 0.74 b,c	200.66 ± 2.87 b
OP10	134.44 ± 2.55 b,f	31.66 ± 0.13 b,f	34.93 ± 0.41 b,c	201.03 ± 1.42 b
OP20	135.29 ± 0.52 f	29.26 ± 1.05 f	33.23 ± 0.1 b	197.79 ± 0.00 b

C-AO = avocado oil; C-VAO = freshly extracted virgin avocado oil; AL5-AL10-AL20, OL5-OL10-OL20, and OP5-OP10-OP20 represent C-VAO enriched with avocado leaves, olive leaves, and olive pomace, respectively, at 5, 10, and 20% (*w/w*) employing ultrasound-assisted maceration. ND = not detected. Results expressed as mean value ± standard deviation (*n* = 3). Different lowercase letters in the same column indicate significant differences (*p* < 0.05) within the whole sample population employing Duncan’s test.

## Data Availability

The original contributions presented in the study are included in the article/Supplementary Material, further inquiries can be directed to the corresponding author.

## Supplementary Materials

The following supporting information can be downloaded at https://www.mdpi.com/article/10.3390/foods14020294/s1, Table S1: Quality characteristics and physicochemical composition of the control extra virgin olive oil sample; Table S2: Fatty acid profile and oxidative susceptibility of control and enriched oil samples; Table S3: Color parameters of the studied control and enriched virgin avocado oils; Table S4: Volatile compounds of the control and enriched virgin avocado oils; Figure S1: High-resolution mass spectrum of avocado leaf extract; Figure S2: ATR-FT-IR spectra of the studied control and enriched virgin avocado oils; Figure S3: Scattered t1/t2 score plots of the studied control and enriched virgin avocado oils; Figure S4: HPLC-DAD chromatograms of the studied control and enriched virgin avocado oils. Figure S5: Pictures of the control and enriched virgin avocado oils. C-VAO = freshly extracted virgin avocado oil; AL5-AL10-AL20, OL5-OL10-OL20, and OP5-OP10-OP20 represent C-VAO enriched with avocado leaves, olive leaves, and olive pomace, respectively, at 5, 10, and 20% (*w/w*) employing ultrasound-assisted maceration.
