# Supplementary material for: Oxidative Stability of Virgin Avocado Oil Enriched with Avocado Leaves and Olive-Fruit-Processing By-Products (Leaves, Pomace) via Ultrasound-Assisted Maceration
Source: Foods. 2025 Jan 17;14(2):294. doi: 10.3390/foods14020294 (PMC11765188; doi:10.3390/foods14020294)
Supplement: Supplementary file 1 [file foods-14-00294-s001.zip › foods-3390426-supplementary.pdf]

SUPPLEMENTARY MATERIAL

Oxidative stability of virgin avocado oil enriched with avocado leaves and olive fruit processing by-products (leaves, pomace) via ultrasound-assisted maceration

Ioanna Pyrka [1], Stavros Stefanidis [1], Stella A. Ordoudi [2,3], Sofia Lalou [4], Nikolaos Nenadis [1,3*]

[1] Laboratory of Food Chemistry and Technology, School of Chemistry, Aristotle University of Thessaloniki, 54124 Thessaloniki, Greece; ioannapyrka@chem.auth.gr (I.P.); stavstef@chem.auth.gr (S.S.)
[2] Laboratory of Oenology and Alcoholic Beverages, Department of Food Science and Technology, School of Agriculture, , Aristotle University of Thessaloniki, 54124 Thessaloniki, Greece; steord@agro.auth.gr
[3] Natural Products Research Centre of Excellence-AUTH (NatPro-AUTH), Center for Interdisciplinary Research and Innovation (CIRI-AUTH), 57001 Thessaloniki, Greece
[4] Department of Food Science and Technology, Perrotis College, American Farm School, 55102 Thessaloniki, Greece; slalou@afs.edu.gr
* Correspondence: niknen@chem.auth.gr

Table S1. Quality characteristics and physicochemical composition of the control extra virgin olive oil sample.

Table S2. Fatty acid profile and oxidative susceptibility of control and enriched oil samples.

Table S3. Color parameters of the studied control and enriched virgin avocado oils.

Table S4. Volatile compounds of the control and enriched virgin avocado oils.

Figure S1. High-resolution mass spectrum of avocado leaf extract.

Figure S2. ATR-FT-IR spectra of the studied control and enriched virgin avocado oils.

Figure S3. Scattered t1/t2 score plots of the studied control and enriched virgin avocado oils.

Figure S4. HPLC-DAD chromatograms of the studied control and enriched virgin avocado oils.

Figure S5. Pictures of the control and enriched virgin avocado oils.

Table S1. Quality characteristics and physicochemical composition of the control extra virgin olive oil sample (C-EVOO).

Quality Parameters	
Free Acidity (% oleic acid)	0.45 ± 0.04
Peroxide value (meq O_2/kg oil)	9.27 ± 0.58
K232	1.60 ± 0.02
K270	0.11 ± 0.01
Oxidative Stability	
Induction period (h)	35.00 ± 0.64
Predicted induction period (months, 20 °C)	24.89 ± 0.46
Antioxidant Activity *(mmol TE/kg oil)*	
DPPH• (oil)	1.52 ± 0.02
DPPH• (oil polar extract)	1.14 ± 0.02
CUPRAC (oil polar extract)	2.34 ± 0.12
Fatty acids	
Myristic acid (C14:0)	0.01 ± 0.00
Palmitic acid (C16:0)	12.86 ± 0.07
Palmitoleic acid (C16:1)	0.29 ± 0.00
Margaric acid (C17:0)	0.06 ± 0.01
Heptadecenoic acid (C17:1)	0.07 ± 0.00
Stearic acid (C18:0)	1.45 ± 0.03
Oleic acid (C18:1 cis)	78.44 ± 0.01
Linoleic acid (C18:2 cis-cis)	5.59 ± 0.01
Linolenic acid (C18:3 cis)	0.42 ± 0.01
Arachidic acid (C20:0)	0.33 ± 0.02
Gadoleic acid (C20:1)	0.12 ± 0.00
Lignoceric acid (C24:0)	0.35 ± 0.01
SFA	15.07
MUFA	78.91
PUFA	6.01
TUFA	84.93
PUFA/SFA	0.40
TUFA/SFA	5.64
OS	372.63
Phenolic Content *(mg/kg oil)*	
TPC	348.77 ± 5.87
Hydroxytyrosol	152.00 ± 1.00
Tyrosol	372.00 ± 1.00
Tocopherols *(mg/kg oil)*	
γ-Tocopherol	4.70 ± 0.23
α-Tocopherol	239.91 ± 0.72
Pigments *(mg/kg oil)*	
Total Chlorophylls	2.43 ± 0.00
Total Carotenoids	1.17 ± 0.00
β-Carotene	2.33 ± 0.05
Lutein	2.60 ± 0.03
α-Pheophytin	14.01 ± 0.08

Color	
L^*	54.16 ± 0.04
a^*	-1.67 ± 0.03
b^*	55.12 ± 0.37
h	91.74 ± 0.05
C^*	55.15 ± 0.37
VOCs (%)	
Acids	
4-Acetylbenzoic acid	2.52 ± 0.71
4-Formylbenzoic acid	2.38 ± 1.01
Nonanoic acid	2.92 ± 0.55
Octanoic acid	1.68 ± 0.50
Alcohols	
1-Octanol	0.83 ± 0.00
1-Penten-3-ol	0.53 ± 0.01
2-Penten-1-ol	0.65 ± 0.10
Aldehydes	
2-Hexenal	17.96 ± 0.14
3-Hexenal	2.46 ± 0.41
4-Oxohex-2-enal	4.58 ± 0.13
Decanal	0.81 ± 0.03
Hexanal	1.72 ± 0.03
Nonanal	8.55 ± 0.40
Pentanal	1.08 ± 0.05
Esters	
(E)-3-Hexen-1-ol acetate	8.25 ± 0.16
Acetic acid octyl ester	1.6 ± 0.00
Benzoic acid undecyl ester	1.07 ± 0.04
Hydrocarbons	
(E)-2,6-Dimethyl-1,3,5,7-octatetraene	6.09 ± 0.22
(E)-5-Octadecene	15.04 ± 2.24
3-Ethyl-1,5-octadiene	1.12 ± 0.48
5-Methyl-5-propyl-nonane	1.04 ± 0.04
a-Farnesene	2.51 ± 0.07
b-Neoclovene	1.21 ± 0.11
Copaene	1.57 ± 0.03
Tridecane	6.61 ± 0.33
Ketones	
2,2-Dimethyl-3-heptanone	1.34 ± 0.08
6-Methyl-5-hepten-2-one	0.82 ± 0.13
Penten-3-one	1.79 ± 0.05
Others	
Methoxy-phenyl-oxime	1.28 ± 0.77

Results expressed as mean value ± standard deviation (n = 3).

Table S2. Fatty acid profile and oxidative susceptibility of control and enriched oil samples.

Fatty acids		C-AO	C-VAO	AL5	AL10	AL20	OL5	OL10	OL20	OP5	OP10	OP20
Myristic acid	C14:0	0.03 ± 0.00^{c,d,e}	0.03 ± 0.00^{b,c,d}	0.04±0.00^{e}	0.03±0.00^{c,d}	0.03±0.00^{b,c,d}	0.03±0.00^{b,c}	0.03±0.00^{b,c,d}	0.03±0.00^{d,e}	0.03±0.00^{b,c,d}	0.03±0.00^{b}	0.03±0.00^{b,c,d}
Palmitic acid	C16:0	15.98 ± 0.23^{c}	15.42 ± 0.18^{b,c}	15.65±0.15^{b,c}	15.29±0.04^{b}	15.64±0.05^{b,c}	15.76±0.00^{b,c}	15.47±0.09^{b,c}	15.50±0.25^{b,c}	15.63±0.10^{b,c}	15.31±0.36^{b}	15.42±0.09^{b,c}
Palmitoleic acid	C16:1	3.92 ± 0.04^{b}	4.78 ± 0.04^{e}	4.50±0.04^{c,d}	4.41±0.01^{c,d}	4.79±0.03^{e}	4.56±0.06^{c,d}	4.39±0.04^{c}	4.76±0.13^{e}	4.45±0.03^{c}	4.37±0.09^{c}	4.65±0.01^{d,e}
Margaric acid	C17:0	0.03 ± 0.00^{a}	0.02 ± 0.00^{a}	0.03±0.00^{a}	0.03±0.00^{a}	0.02±000^{a}	0.02±0.00^{a}	0.02±0.00^{a}	0.02±0.00^{a}	0.02±0.00^{a}	0.03±0.00^{a}	0.02±0.00^{a}
Heptadecenoic acid	C17:1	0.09 ± 0.00^{b}	0.10 ± 0.01^{b,c}	0.13±0.00^{g,h}	0.14±0.00^{h}	0.10±0.00^{c,d}	0.11±0.00^{d,e,f}	0.13±0.00^{f,g,h}	0.10±0.00^{c,d,e}	0.11±0.01^{d,e,f}	0.12±0.00^{e,f,g}	0.09±0.00^{b,c}
Stearic acid	C18:0	0.53 ± 0.04^{d}	0.28 ± 0.01^{a,b}	0.32±0.00^{a,b,c}	0.32±0.01^{a,b,c}	0.28±0.00^{a,b}	0.30±0.00^{a,b,c}	0.34±0.01^{c}	0.28±0.01^{a,b}	0.32±0.01^{b,c}	0.33±0.03^{c}	0.28±0.00^{a}
Oleic acid	C18:1 cis	69.44 ± 0.20^{a}	70.38 ± 0.01^{b,c}	70.91±0.09^{d,e}	71.16±0.13^{e,f}	70.07±0.01^{b}	71.09±0.09^{e,f}	70.89±0.05^{d,e}	70.16±0.24^{b}	71.39±0.08^{f}	71.33±0.10^{f}	70.58±0.11^{c,d}
Linoleic acid	C18:2 cis-cis	9.28 ± 0.05^{g}	8.52 ± 0.11^{f}	7.99±0.22^{c,d}	8.12±0.08^{c,d,e}	8.51±0.00^{f}	7.76±0.05^{b,c}	8.26±0.10^{d,e,f}	8.58±0.09^{f}	7.64±0.02^{b}	8.03±0.26^{c,d}	8.42±0.01^{e,f}
Linolenic acid	C18:3 cis	0.46 ± 0.01^{e}	0.32 ± 0.04^{b,c}	0.23±0.04^{a,b}	0.26±0.01^{a,b,c}	0.34±0.00^{c,d}	0.18±0.00^{a}	0.28±0.00^{a,b,c}	0.35±0.02^{c,d}	0.22±0.07^{a,b}	0.24±0.04^{a,b}	0.30±0.02^{b,c}
Arachidic acid	C20:0	0.07 ± 0.00^{b}	0.03±0.00^{a}	0.03±0.00^{a}	0.04±0.00^{a}	0.02±0.00^{a}	0.03±0.00^{a}	0.03±0.00^{a}	0.02±0.00^{a}	0.02±0.00^{a}	0.03±0.00^{a}	0.03±0.00^{a}
Gadoleic acid	C20:1	0.09 ± 0.01^{a}	0.10 ± 0.01^{a}	0.10±0.01^{a}	0.12±0.01^{a}	0.11±0.00^{a}	0.09±0.00^{a}	0.10±0.01^{a}	0.11±0.00^{a}	0.09±0.03^{a}	0.11±0.01^{a}	0.11±0.01^{a}
Lignoceric acid	C24:0	0.08 ± 0.00^{c}	0.05 ± 0.00^{a}	0.08±0.01^{c}	0.08±0.00^{c}	0.08±0.00^{c}	0.07±0.00^{c}	0.06±0.00^{a,b}	0.08±0.01^{c}	0.07±0.00^{a,b}	0.08±0.01^{c}	0.07±0.00^{b,c}
SFA		16.73	15.86	16.14	15.80	16.08	16.21	15.96	15.94	16.10	15.81	15.85
MUFA		73.53	75.32	75.64	75.82	75.06	75.85	75.51	75.13	76.04	75.92	75.43
PUFA		9.74	8.83	8.22	8.38	8.85	7.94	8.54	8.93	7.86	8.27	8.72
TUFA		83.27	84.15	83.86	84.20	83.92	83.79	84.04	84.06	83.90	84.19	84.15
SFA/MUFA		0.23	0.21	0.21	0.21	0.21	0.21	0.21	0.21	0.21	0.21	0.21
SFA/TUFA		0.20	0.19	0.19	0.19	0.19	0.19	0.19	0.19	0.19	0.19	0.19
OS		537.22	490.16	458.23	467.49	492.41	442.84	474.92	496.12	442.01	460.88	484.02

C-AO = avocado oil; C-VAO = virgin avocado oil; AL, OL, and OP = C-VAO enriched with avocado leaves, olive leaves, and olive pomace, respectively, at 5, 10, and 20% (w/w) employing ultrasound assisted maceration; ND = not detected; SFA = saturated, MUFA = monounsaturated, PUFA = polyunsaturated, and TUFA = total unsaturated free fatty acids; OS = oxidative susceptibility. Values are expressed as relative peak area (%) = 100 *x* (peak area of individual fatty acid / total peak area of identified fatty acids. Results expressed as mean value ± standard deviation (n = 3). Means followed by different superscript letter in the same row showed significant difference ($p < 0.05$) within the whole sample population by Duncan's test.

Table S3. Color parameters of the studied control and enriched virgin avocado oils.

Sample	L*	a*	b*	ΔE	C*	h
C-AO	49.09 ± 0.04 b	-0.24 ± 0.01 b	72.21 ± 0.39 b	-	72.21 ± 0.39 b	90.19 ± 0.01 b
C-VAO	50.87 ± 0.07 c	-2.82 ± 0.05 c	74.26 ± 0.50	-	74.31 ± 0.49 c	92.18 ± 0.05 c
AL5	30.18 ± 0.59 d	-10.90 ± 0.09 d	47.84 ± 0.94 d	34.51 ± 1.05 a	49.07 ± 0.93 d	102.84 ± 0.19 d
AL10	16.44 ± 0.42 e	-9.09 ± 0.11 e	26.31 ± 0.83 e	59.36 ± 0.91 b	27.84 ± 0.82 e	109.07 ± 0.41 e
AL20	4.91 ± 0.05 f	-0.64 ± 0.07 f	7.62 ± 0.01 f	80.98 ± 0.02 c	7.64 ± 0.02 f	94.78 ± 0.53 f
OL5	41.40 ± 0.06 g	-6.22 ± 0.04 g	66.46 ± 0.34 g, j	12.73 ± 0.24 d	66.75 ± 0.34 g, j	95.35 ± 0.01 g
OL10	34.87 ± 0.21 h	-8.64 ± 0.05 h	57.32 ± 0.56 h	24.01 ± 0.53 e	57.97 ± 0.55 h	98.57 ± 0.12 h
OL20	25.58 ± 0.19 i	-9.95 ± 0.04 i	42.24 ± 0.18 i	41.42 ± 0.17 f	43.40 ± 0.18 i	103.25 ± 0.06 i
OP5	45.54 ± 0.12 j	0.97 ± 0.02 j	66.13 ± 0.40 g	10.47 ± 0.28 g, h	66.13 ± 0.40 g, j	89.20 ± 0.12 j
OP10	43.31 ± 0.07 k	0.73 ± 0.06 k	66.47 ± 0.12 g, j	11.42 ± 0.07 h	66.47 ± 0.12 g	89.31 ± 0.10 j, k
OP20	44.59 ± 0.07 l	0.35 ± 0.03 l	67.47 ± 1.78 j	9.80 ± 1.28 g	67.47 ± 1.78 j	89.61 ± 0.14 k

C-AO = avocado oil; C-VAO = virgin avocado oil; AL, OL, and OP = C-VAO enriched with avocado leaves, olive leaves, and olive pomace, respectively, at 5, 10, and 20% (w/w) employing ultrasound assisted maceration. Results expressed as mean value ± standard deviation (n = 3). Values followed by different lowercase letter in the same column showed significant difference ($p < 0.05$) within the whole sample population by the Duncan test

Table S4. Volatile compounds in the control and enriched virgin avocado oils.

	Compound	C-EVOO	C-AO	C-VAO	AL5	AL10	AL20	OL5	OL10	OL20	OP5	OP10	OP20
	**Acids**												
1	2-Ethylhexanoic acid	*ND*	*ND*	*ND*	*ND*	*ND*	*ND*	1.58^{a}	3.85^{b}	*ND*	*ND*	1.92^{a}	*ND*
2	4-Acetylbenzoic acid	2.52^{a,b}	*ND*	5.89^{b,c}	1.60^{a}	1.52^{a}	0.94^{a}	4.01^{a,b,c}	6.95^{c}	14.67^{d}	3.33^{a,b,c}	2.94^{a,b,c}	1.13^{a}
3	4-Formylbenzoic acid	2.38^{a,b,c,d}	*ND*	6.73^{b,c,d,e}	1.58^{a,b,c,d}	0.95^{a,b,d}	0.71^{a,b}	7.71^{c,e}	7.6^{c,d,e}	4.02^{a,b,c,d,e}	9.84^{e}	3.53^{a,b,c,d,e}	3.79^{a,b,c,d,e}
4	4-Vinylbenzoic acid	*ND*	*ND*	*ND*	*ND*	*ND*	*ND*	*ND*	*ND*	*ND*	1.37	*ND*	*ND*
5	Acetic acid	*ND*	1.56^{a,b}	5.06^{b,c}	*ND*	1.03^{a,b}	0.52^{a}	*ND*	*ND*	21.11^{d}	7.95^{c,e}	11.20^{e,f}	13.65^{f}
6	Benzoic acid	*ND*	0.39^{a}	*ND*	*ND*	0.36^{a}	0.22^{a}	2.72^{b}	2.05^{c,d}	1.76^{c}	2.30^{b,d}	1.48^{c}	1.49^{c}
7	Heptanoic acid	*ND*	*ND*	*ND*	*ND*	*ND*	*ND*	*ND*	*ND*	*ND*	*ND*	0.84^{a}	0.64^{b}
8	Hexanedioic acid	*ND*	*ND*	1.26	*ND*	*ND*	*ND*	*ND*	*ND*	*ND*	*ND*	*ND*	*ND*
9	Hexanoic acid	*ND*	*ND*	1.61^{a,b}	*ND*	0.16^{c}	0.12^{c}	2.61^{d}	1.97^{b,d}	1.06^{a}	*ND*	2.51^{d}	1.64^{a,b}
10	n-Decanoic acid	*ND*	*ND*	*ND*	*ND*	*ND*	*ND*	*ND*	*ND*	*ND*	*ND*	*ND*	1.16
11	Nonanoic acid	2.92^{a}	0.41^{a}	2.07^{a}	0.23^{a}	0.29^{a}	0.18^{a}	3.27^{a}	2.41^{a}	5.24^{a,b}	16.57^{b}	7.30^{a,b}	7.75^{a,b}
12	Octanoic acid	1.68^{a}	*ND*	*ND*	*ND*	0.23^{b}	0.21^{b}	2.38^{a}	2.42^{a}	2.27^{a}	1.93^{a}	5^{c}	*ND*
	Total	*9.50*	*2.35*	*22.62*	*3.41*	*4.54*	*2.90*	*24.29*	*27.26*	*50.14*	*43.29*	*36.71*	*31.24*
	**Alcohols**												
13	(1R,2S,6S,7S,8S)-8-Isopropyl-1-methyl-3-methylenetricyclo [4.4.0.02,7] decane-4-ol	*ND*	1.55^{a}	*ND*	0.68^{b}	0.27^{c}	*ND*	*ND*	*ND*	*ND*	*ND*	*ND*	*ND*
14	1,2,4a,5,6,8a-hexahydro-4,7-dimethyl-1-(1-methylethyl)-Naphthalene	*ND*	0.35	*ND*	*ND*	*ND*	*ND*	*ND*	*ND*	*ND*	*ND*	*ND*	*ND*
15	1-Hexanol	*ND*	0.80^{a,b}	2.11^{c}	0.53^{a,b}	*ND*	*ND*	*ND*	2.62^{c}	1.37^{b}	0.93^{a,b}	0.72^{a,b}	0.57^{a,b}
16	1-Octanol	0.83	*ND*	*ND*	*ND*	*ND*	*ND*	*ND*	*ND*	*ND*	*ND*	*ND*	*ND*
17	1-Penten-3-ol	0.53	*ND*	*ND*	*ND*	*ND*	*ND*	*ND*	*ND*	*ND*	*ND*	*ND*	*ND*
18	2,3,4,4a,5,6,7,8-Octahydro-2-hydroxy-[.alpha./.beta.]-2,4a-dimethyl- Naphthalene	*ND*	*ND*	*ND*	*ND*	*ND*	*ND*	*ND*	*ND*	1.2	*ND*	*ND*	*ND*
19	2-Hexen-1-ol	*ND*	1.52^{a}	4.59^{b}	*ND*	*ND*	*ND*	*ND*	*ND*	*ND*	*ND*	*ND*	*ND*
20	2-Penten-1-ol	0.65	*ND*	*ND*	*ND*	*ND*	*ND*	*ND*	*ND*	*ND*	*ND*	*ND*	*ND*
21	3-Ethyl-phenol	*ND*	*ND*	*ND*	*ND*	*ND*	*ND*	*ND*	*ND*	*ND*	*ND*	*ND*	6.82
22	3-Hexen-1-ol	*ND*	0.37	*ND*	*ND*	*ND*	*ND*	*ND*	*ND*	*ND*	*ND*	*ND*	*ND*

	Compound	C-EVOO	C-AO	C-VAO	AL5	AL10	AL20	OL5	OL10	OL20	OP5	OP10	OP20
23	4-Methyl-1-(1-methylethyl)-bicyclo[3.1.0]hexan-3-ol	*ND*	*ND*	*ND*	*ND*	*ND*	0.39[a]	*ND*	*ND*	*ND*	*ND*	*ND*	0.68[b]
24	Estragole	*ND*	0.20[a]	*ND*	55.31[b]	52.8[c]	49.49[d]	*ND*	*ND*	*ND*	*ND*	*ND*	*ND*
25	Eucalyptol	*ND*	*ND*	*ND*	0.33[a]	0.34[a]	0.33[a]	*ND*	*ND*	*ND*	*ND*	*ND*	*ND*
26	Farnesol	*ND*	*ND*	*ND*	*ND*	*ND*	0.13	*ND*	*ND*	*ND*	*ND*	*ND*	*ND*
27	Linalool	*ND*	*ND*	*ND*	0.15[a]	0.12[b]	0.11[c]	*ND*	*ND*	*ND*	*ND*	*ND*	*ND*
28	Methyleugenol	*ND*	*ND*	*ND*	13.11[a]	15.57[b]	20.17[c]	*ND*	*ND*	*ND*	*ND*	*ND*	*ND*
29	Nerolidol	*ND*	*ND*	*ND*	0.56[a]	0.58[a]	0.78[b]	*ND*	*ND*	*ND*	*ND*	*ND*	*ND*
30	a-Cadinol	*ND*	1.01	*ND*	*ND*	*ND*	*ND*	*ND*	*ND*	*ND*	*ND*	*ND*	*ND*
	Total	*2.02*	*5.80*	*6.70*	*70.67*	*69.68*	*71.41*	*0.00*	*2.62*	*2.57*	*0.93*	*0.72*	*8.07*
	**Aldehydes**												
31	10-Undecenal	*ND*	0.21	*ND*	*ND*	*ND*	*ND*	*ND*	*ND*	*ND*	*ND*	*ND*	*ND*
32	2-Hexenal	17.96[a]	*ND*	20.79[a]	2.73[b,c]	2.03[b,c]	1.40[b]	19.71[a]	*ND*	10.61[d]	7.63[d,e]	6.53[d,e]	5.40[c,e]
33	3-Hexenal	2.46[a]	0.45[b]	6.49[c]	0.56[b]	0.33[b]	*ND*	*ND*	*ND*	*ND*	*ND*	*ND*	*ND*
34	4-Oxohex-2-enal	4.58	*ND*	*ND*	*ND*	*ND*	*ND*	*ND*	*ND*	*ND*	*ND*	*ND*	*ND*
35	a-(2-Methylpropylidene)-benzeneacetaldehyde	*ND*	*ND*	*ND*	*ND*	*ND*	*ND*	3.45	*ND*	*ND*	*ND*	*ND*	*ND*
36	Benzaldehyde	*ND*	*ND*	2.18[a]	0.23[b]	*ND*	*ND*	*ND*	1.78[c]	1.33[d]	1.96[a,c]	*ND*	*ND*
37	Benzeneacetaldehyde	*ND*	*ND*	3.12[a]	0.40[b]	0.25[b]	0.14[b]	4.35[c]	3.81[a]	1.96[d]	1.98[d]	1.46[d]	1.29[d]
38	Cuminaldehyde	*ND*	*ND*	*ND*	*ND*	*ND*	*ND*	*ND*	*ND*	1.19	*ND*	*ND*	*ND*
39	Decanal	0.81[a]	*ND*	1.39[b]	*ND*	*ND*	*ND*	1.51[b]	1.5[b]	*ND*	*ND*	*ND*	*ND*
40	Heptanal	*ND*	*ND*	2.82	*ND*	*ND*	*ND*	*ND*	*ND*	*ND*	*ND*	*ND*	*ND*
41	Hexanal	1.72[a,b]	*ND*	1.50[a,b]	*ND*	*ND*	0.24[a]	5.44[c]	24.16[d]	2.83[b]	4.68[c]	2.14[b]	1.81[a,b]
42	Nonanal	8.55[a]	1.02[b]	2.16[b,c]	0.33[b]	0.16[b]	0.10[b]	3.82[b,c]	*ND*	2.06[b]	6.45[a,c]	1.12[b]	1.31[b]
43	Octanal	*ND*	*ND*	*ND*	*ND*	*ND*	*ND*	*ND*	*ND*	*ND*	*ND*	*ND*	7.08
44	Pentanal	1.08[a]	0.75[b]	*ND*	0.28[c]	0.20[c,d]	*ND*	1.94[e]	1.94[e]	*ND*	*ND*	*ND*	*ND*
45	Tetradecanal	*ND*	*ND*	2.74	*ND*	*ND*	*ND*	*ND*	*ND*	*ND*	*ND*	*ND*	*ND*
	Total	*37.16*	*2.42*	*43.19*	*4.54*	*2.96*	*1.88*	*40.22*	*33.19*	*19.99*	*22.70*	*11.26*	*16.90*
	**Esters**												
46	(E)-3-Hexen-1-ol acetate	8.25	*ND*	*ND*	*ND*	*ND*	*ND*	*ND*	*ND*	*ND*	*ND*	*ND*	*ND*
47	(E)-4-Tridecen-1-ol acetate	*ND*	*ND*	*ND*	*ND*	*ND*	0.09	*ND*	*ND*	*ND*	*ND*	*ND*	*ND*
48	1-Norbornanemethanol acetate	*ND*	*ND*	*ND*	*ND*	0.19[a]	0.23[b]	*ND*	*ND*	*ND*	*ND*	*ND*	*ND*

	Compound	C-EVOO	C-AO	C-VAO	AL5	AL10	AL20	OL5	OL10	OL20	OP5	OP10	OP20
49	2-Hexen-1-ol acetate	*ND*	2.02	*ND*	*ND*	*ND*	*ND*	*ND*	*ND*	*ND*	*ND*	*ND*	*ND*
50	4-Acetylbenzoic acid methyl ester	*ND*	*ND*	*ND*	*ND*	*ND*	*ND*	*ND*	*ND*	1.20	*ND*	*ND*	*ND*
51	4-Formyl-benzoic acid methyl ester	*ND*	*ND*	*ND*	*ND*	*ND*	*ND*	*ND*	*ND*	1.41^{b}	1.08^{a}	1.32^{b}	1.73^{c}
52	Acetic acid hexyl ester	*ND*	2.32	*ND*	*ND*	*ND*	*ND*	*ND*	*ND*	*ND*	*ND*	*ND*	*ND*
53	Acetic acid octyl ester	1.6^{a}	0.24^{b}	*ND*	*ND*	*ND*	*ND*	*ND*	*ND*	*ND*	*ND*	*ND*	*ND*
54	Acetic acid 1,7,7-trimethyl-bicyclo[2.2.1]hept-2-yl ester	*ND*	0.30	*ND*	*ND*	*ND*	*ND*	*ND*	*ND*	*ND*	*ND*	*ND*	*ND*
55	Acetic acid 3,4-dihydroxy-3-methyl-butyl ester	*ND*	0.79	*ND*	*ND*	*ND*	*ND*	*ND*	*ND*	*ND*	*ND*	*ND*	*ND*
56	Benzoic acid undecyl ester	1.07^{a}	*ND*	3.42^{b}	*ND*	*ND*	*ND*	2.35^{c}	1.57^{a}	4.90^{d}	*ND*	2.00^{c}	*ND*
57	Dodec-(5Z)-enyl acetate	*ND*	0.76	*ND*	*ND*	*ND*	*ND*	*ND*	*ND*	*ND*	*ND*	*ND*	*ND*
58	Geranyl isobutyrate	*ND*	*ND*	*ND*	*ND*	*ND*	0.25	*ND*	*ND*	*ND*	*ND*	*ND*	*ND*
59	Hexadecanoic acid methyl ester	*ND*	*ND*	2.75	*ND*	*ND*	*ND*	*ND*	*ND*	*ND*	*ND*	*ND*	*ND*
	Total	*10.92*	*6.43*	*6.18*	*0.00*	*0.19*	*0.57*	*2.35*	*1.57*	*7.51*	*1.08*	*3.32*	*1.73*
	**Hydrocarbons**												
60	(1R,3aS,8aS)-7-Isopropyl-1,4-dimethyl-1,2,3,3a,6,8a-hexahydroazulene	*ND*	*ND*	*ND*	0.58^{a}	*ND*	*ND*	2.94^{b}	2.4^{c}	*ND*	1.11^{d}	0.98^{a,d}	1.22^{d}
61	(1S)-6,6-Dimethyl-2-methylene-Bicyclo[3.1.1]heptane	*ND*	*ND*	*ND*	*ND*	0.69^{a}	0.91^{b}	*ND*	*ND*	*ND*	*ND*	*ND*	*ND*
62	(3E, 6Z)-3,7,11-Trimethyl-1,3,6,10-dodecatetraene	*ND*	*ND*	*ND*	*ND*	0.16	*ND*	*ND*	*ND*	*ND*	*ND*	*ND*	*ND*
63	(E)-a-Bisabolene	*ND*	*ND*	*ND*	*ND*	*ND*	*ND*	*ND*	*ND*	*ND*	*ND*	0.74	*ND*
64	(E)-2,6-Dimethyl-1,3,5,7-octatetraene	6.09^{a}	0.29^{b}	*ND*	*ND*	*ND*	*ND*	*ND*	*ND*	*ND*	*ND*	*ND*	*ND*
65	(E)-5-Octadecene	15.04^{a}	0.41^{b}	*ND*	*ND*	*ND*	*ND*	*ND*	*ND*	*ND*	*ND*	*ND*	*ND*
66	(E)-Caryophyllene	*ND*	8.95^{a,b,c,d}	7.70^{a,b,c,d,e}	10.50^{c,d,f}	9.77^{b,c,d}	10.10^{c,d,f}	15.80^{f}	13.72^{d,f}	6.07^{a,b,c,e,g}	4.99^{a,b,e,g}	4.18^{a,e,g}	3.47^{e,g}
67	(E)-Cycloundecene	*ND*	*ND*	*ND*	*ND*	*ND*	*ND*	1.49	*ND*	*ND*	*ND*	*ND*	*ND*
68	(S,1Z,6Z)-8-Isopropyl-1-methyl-5-methylenecyclodeca-1,6-diene	*ND*	0.45^{a,b}	*ND*	*ND*	0.47^{b}	0.42^{a}	*ND*	*ND*	*ND*	*ND*	*ND*	*ND*
69	(Z)-3,7-Dimethyl-1,3,6-octatriene	*ND*	7.31^{a}	*ND*	0.31^{b}	0.3^{b}	*ND*	*ND*	*ND*	*ND*	*ND*	*ND*	*ND*

	Compound	C-EVOO	C-AO	C-VAO	AL5	AL10	AL20	OL5	OL10	OL20	OP5	OP10	OP20
70	1,13-Tetradecadiene	*ND*	0.56	*ND*	*ND*	*ND*	*ND*	*ND*	*ND*	*ND*	*ND*	*ND*	*ND*
71	1-Methyl-3-methylene-8-(1-methylethyl)-tricyclo[4.4.0.0(2,7)]decane	*ND*	0.18	*ND*	*ND*	*ND*	*ND*	*ND*	*ND*	*ND*	*ND*	*ND*	*ND*
72	2,2,4-Trimethylpentane	*ND*	*ND*	*ND*	*ND*	*ND*	*ND*	*ND*	*ND*	7	*ND*	*ND*	*ND*
73	2,6-Dimethyl-2,4,6-octatriene	*ND*	0.74	*ND*	*ND*	*ND*	*ND*	*ND*	*ND*	*ND*	*ND*	*ND*	*ND*
74	2-Methylene-4,8,8-trimethyl-4-vinyl-bicyclo[5.2.0]nonane	*ND*	*ND*	*ND*	*ND*	0.31	*ND*	*ND*	*ND*	*ND*	*ND*	*ND*	*ND*
75	3-Ethyl-1,5-octadiene	1.12	*ND*	*ND*	*ND*	*ND*	*ND*	*ND*	*ND*	*ND*	*ND*	*ND*	*ND*
76	3-Methyl-6-(1-methylethylidene)-cyclohexene	*ND*	0.25	*ND*	*ND*	*ND*	*ND*	*ND*	*ND*	*ND*	*ND*	*ND*	*ND*
77	4-(1,5-Dimethyl-1,4-hexadienyl)-1-methyl-cyclohexene	*ND*	*ND*	1.74	*ND*	*ND*	*ND*	*ND*	*ND*	*ND*	*ND*	*ND*	*ND*
78	4-Carene	*ND*	5.35	*ND*	*ND*	*ND*	*ND*	*ND*	*ND*	*ND*	*ND*	*ND*	*ND*
79	4-Methylene-1-(1-methylethyl)-bicyclo[3.1.0]hexane	*ND*	*ND*	*ND*	0.39	0.38^{a}	*ND*	*ND*	*ND*	*ND*	*ND*	*ND*	*ND*
80	5-Methyl-3-(1-methylethylidene)-1,4-hexadiene	*ND*	*ND*	*ND*	*ND*	*ND*	0.10	*ND*	*ND*	*ND*	*ND*	*ND*	*ND*
81	5-Methyl-5-propyl-nonane	1.04	*ND*	*ND*	*ND*	*ND*	*ND*	*ND*	*ND*	*ND*	*ND*	*ND*	*ND*
82	a-Calacorene	*ND*	1.08	*ND*	*ND*	*ND*	*ND*	*ND*	*ND*	*ND*	*ND*	*ND*	*ND*
83	a-Cubebene	*ND*	4.66^{a}	*ND*	*ND*	0.49^{b}	0.69^{b}	*ND*	*ND*	*ND*	*ND*	*ND*	*ND*
84	a-Farnesene	2.51^{a}	*ND*	*ND*	*ND*	*ND*	0.3^{b}	*ND*	*ND*	1.65^{c}	*ND*	*ND*	*ND*
85	a-Humulene	*ND*	1.51^{a,b}	*ND*	1.11^{a,c}	1.07^{a,c}	1.21^{a,b,c}	1.80^{b}	1.72^{a,b}	*ND*	1.17^{a,c}	*ND*	0.74^{c}
86	a-Phellandrene	*ND*	3.60^{a}	*ND*	*ND*	0.17^{b}	*ND*	*ND*	*ND*	*ND*	*ND*	*ND*	*ND*
87	a-Pinene	*ND*	1.92^{a}	*ND*	0.21^{b}	0.27^{b,c}	0.33^{c}	*ND*	*ND*	*ND*	*ND*	*ND*	*ND*
88	b-Cubebene	*ND*	1.27^{a}	*ND*	*ND*	*ND*	0.28^{a}	*ND*	*ND*	*ND*	*ND*	*ND*	*ND*
89	b-Myrcene	*ND*	4.39^{a}	*ND*	0.56^{b}	0.42^{b,c}	1.03^{d}	*ND*	*ND*	*ND*	*ND*	*ND*	*ND*
90	b-Neoclovene	1.21	*ND*	*ND*	*ND*	*ND*	*ND*	*ND*	*ND*	*ND*	*ND*	*ND*	*ND*
91	b-Ocimene	*ND*	1.9^{a}	*ND*	*ND*	*ND*	0.31^{b}	*ND*	*ND*	*ND*	*ND*	*ND*	*ND*
92	b-Pinene	*ND*	1.54^{a}	*ND*	0.73^{b}	*ND*	*ND*	*ND*	*ND*	*ND*	*ND*	*ND*	*ND*
93	Cadinene	*ND*	3.29^{a}	*ND*	0.76^{b}	1.12^{b}	*ND*	*ND*	*ND*	*ND*	*ND*	*ND*	*ND*
94	Camphene	*ND*	0.49	*ND*	*ND*	*ND*	*ND*	*ND*	*ND*	*ND*	*ND*	*ND*	*ND*
95	cis-Calamenene	*ND*	0.81^{a}	*ND*	0.28^{b,c}	0.45^{b}	*ND*	*ND*	*ND*	*ND*	*ND*	*ND*	*ND*

	Compound	C-EVOO	C-AO	C-VAO	AL5	AL10	AL20	OL5	OL10	OL20	OP5	OP10	OP20
96	Copaene	1.57^{a}	2.86^{b}	2.90^{b}	1.76^{a}	1.41^{a}	1.36^{a}	3.85^{c}	$3.40^{b,c}$	1.88^{a}	1.55^{a}	1.26^{a}	1.12^{a}
97	Cyclosativene	*ND*	*ND*	*ND*	0.44	*ND*	*ND*	*ND*	*ND*	*ND*	*ND*	*ND*	*ND*
98	Decane	*ND*	*ND*	*ND*	*ND*	*ND*	*ND*	*ND*	*ND*	*ND*	*ND*	9.35^{a}	6.88^{b}
99	Dodecane	*ND*	*ND*	2.97^{a}	*ND*	*ND*	*ND*	*ND*	$1.58^{b,c}$	0.93^{b}	$2.18^{c,d}$	$2.56^{a,d}$	3.9^{e}
100	g-Amorphene	*ND*	*ND*	*ND*	*ND*	0.16^{a}	0.20^{b}	*ND*	*ND*	*ND*	*ND*	*ND*	*ND*
101	g-Elemene	*ND*	0.22	*ND*	*ND*	*ND*	*ND*	*ND*	*ND*	*ND*	*ND*	*ND*	*ND*
102	Germacrene D	*ND*	10.44^{b}	$1.95^{c,d,e,f}$	$1.16^{a,c,d,e}$	$1.08^{a,c,d}$	$1.4^{c,d,e}$	$2.5^{e,f}$	3.48^{f}	$1.17^{a,c,d}$	$2.45^{d,e,f}$	$0.9^{a,c}$	$0.76^{a,c}$
103	g-Muurolene	*ND*	1.53^{a}	*ND*	*ND*	0.17^{b}	1.12^{c}	*ND*	*ND*	*ND*	*ND*	*ND*	*ND*
104	g-Terpinene	*ND*	0.18^{a}	*ND*	0.16^{b}	*ND*	0.1^{c}	*ND*	*ND*	*ND*	*ND*	*ND*	*ND*
105	Hexane	*ND*	*ND*	1.80^{a}	0.93^{b}	0.72^{c}	0.18^{d}	*ND*	*ND*	*ND*	*ND*	*ND*	*ND*
106	Isogermacrene D	*ND*	1.39^{a}	*ND*	*ND*	*ND*	0.52^{b}	*ND*	*ND*	*ND*	*ND*	*ND*	*ND*
107	Limonene	*ND*	5.86^{a}	*ND*	$0.25^{b,c}$	0.46^{b}	$0.18^{b,c}$	*ND*	*ND*	*ND*	*ND*	*ND*	*ND*
108	Octahydro-1,7a-dimethyl-5-(1-methylethyl)-[1S-(1α,2α,3αβ,4α,5α,7αβ,8S*)]-1,2,4-Metheno-1H-indene	*ND*	*ND*	*ND*	*ND*	0.75^{a}	0.28^{b}	*ND*	*ND*	*ND*	*ND*	*ND*	*ND*
109	p-Cymene	*ND*	6.96	*ND*	*ND*	*ND*	*ND*	*ND*	*ND*	*ND*	*ND*	*ND*	*ND*
110	Tetradecane	*ND*	*ND*	*ND*	*ND*	*ND*	*ND*	*ND*	*ND*	*ND*	1.88^{a}	1.13^{b}	2.03^{a}
111	trans-a-Bergamotene	*ND*	0.87	*ND*	*ND*	*ND*	*ND*	*ND*	*ND*	*ND*	*ND*	*ND*	*ND*
112	trans-Calamenene	*ND*	*ND*	*ND*	*ND*	*ND*	0.35	*ND*	*ND*	*ND*	*ND*	*ND*	*ND*
113	Tridecane	6.61^{a}	*ND*	*ND*	*ND*	*ND*	*ND*	2.89^{b}	2.41^{c}	1.1^{d}	*ND*	1.06^{d}	1.04^{d}
	Total	*35.17*	*81.27*	*19.05*	*20.12*	*20.83*	*21.38*	*31.27*	*28.71*	*19.79*	*15.34*	*22.16*	*21.17*
	**Ketones**												
114	(Z)-Undec-6-en-2-one	*ND*	*ND*	*ND*	0.39^{a}	0.38^{a}	0.45^{b}	*ND*	*ND*	*ND*	*ND*	*ND*	*ND*
115	1'-Hydroxy-4,3'-dimethyl-bicyclohexyl-3,3'-dien-2-one	*ND*	*ND*	*ND*	*ND*	*ND*	*ND*	*ND*	*ND*	*ND*	*ND*	1.17^{a}	1.36^{b}
116	2,2-Dimethyl-3-heptanone	1.34	*ND*	*ND*	*ND*	*ND*	*ND*	*ND*	*ND*	*ND*	*ND*	*ND*	*ND*
117	2,3-Octanedione	*ND*	*ND*	*ND*	*ND*	*ND*	*ND*	*ND*	4.84^{a}	*ND*	*ND*	5.41^{a}	*ND*
118	2-Methyl-1-hepten-6-one	*ND*	*ND*	*ND*	*ND*	*ND*	*ND*	*ND*	*ND*	*ND*	*ND*	*ND*	3.28
119	2-Methyl-3-octanone	*ND*	*ND*	*ND*	*ND*	*ND*	*ND*	*ND*	*ND*	*ND*	9.45	*ND*	*ND*
120	2-Nonanone	*ND*	*ND*	*ND*	0.48^{a}	0.44^{b}	0.42^{b}	*ND*	*ND*	*ND*	*ND*	*ND*	*ND*
121	2-Undecanone	*ND*	*ND*	*ND*	*ND*	0.78^{a}	0.78^{a}	*ND*	*ND*	*ND*	*ND*	*ND*	*ND*

	Compound	C-EVOO	C-AO	C-VAO	AL5	AL10	AL20	OL5	OL10	OL20	OP5	OP10	OP20
122	3-(1-Phenyl-ethoxy)-3H-isobenzofuran-1-one	*ND*	*ND*	2.26^{a}	*ND*	*ND*	0.12^{b}	*ND*	*ND*	*ND*	*ND*	*ND*	*ND*
123	4-Hydroxy-3-methyl-2-butanone	*ND*	*ND*	*ND*	*ND*	*ND*	*ND*	*ND*	*ND*	*ND*	*ND*	10.9	*ND*
124	5-Hydroxy-9-oxabicyclo[3.3.1]nonan-2-one	*ND*	*ND*	*ND*	*ND*	*ND*	*ND*	*ND*	*ND*	*ND*	5.33^{a}	3.68^{b}	6.27^{c}
125	6-Methyl-5-hepten-2-one	0.82	*ND*	*ND*	*ND*	*ND*	*ND*	*ND*	*ND*	*ND*	*ND*	*ND*	*ND*
126	Butyrolactone	*ND*	*ND*	*ND*	*ND*	*ND*	*ND*	*ND*	*ND*	*ND*	1.00^{a}	1.38^{b}	2.18^{c}
127	Penten-3-one	1.79	*ND*	*ND*	*ND*	*ND*	*ND*	*ND*	*ND*	*ND*	*ND*	*ND*	*ND*
	Total	*3.95*	*0.00*	*2.26*	*0.87*	*1.60*	*1.78*	*0.00*	*4.84*	*0.00*	*15.78*	*22.53*	*13.10*
	**Others**												
128	2,3-Dihydro-benzofuran	*ND*	0.62^{a}	*ND*	*ND*	*ND*	*ND*	*ND*	*ND*	*ND*	*ND*	0.78^{b}	1.34^{c}
129	3-Ethenyl-pyridine	*ND*	*ND*	*ND*	*ND*	*ND*	*ND*	*ND*	*ND*	*ND*	*ND*	*ND*	2.14
130	5-Ethenyl-2-methyl-pyridine	*ND*	*ND*	*ND*	*ND*	*ND*	*ND*	*ND*	*ND*	*ND*	*ND*	1.28^{a}	3.50^{b}
131	Caryophyllene oxide	*ND*	0.65	*ND*	*ND*	*ND*	*ND*	*ND*	*ND*	*ND*	*ND*	*ND*	*ND*
132	Methoxy-phenyl-oxime	1.28^{a,b}	0.46^{c,d}	*ND*	0.39^{c,d}	0.19^{c,d}	0.09^{c}	1.86^{a}	1.81^{a}	*ND*	0.87^{b,d}	1.23^{a,b}	0.81^{b,d}
	Total	*1.28*	*1.74*	*0.00*	*0.39*	*0.19*	*0.09*	*1.86*	*1.81*	*0.00*	*0.87*	*3.30*	*7.79*

C-AO = avocado oil, C-VAO = virgin avocado oil, AL, OL and OP = C-VAO enriched with avocado leaves, olive leaves and olive pomace, respectively, at 5, 10 and 20% (w/w) employing ultrasound assisted maceration. ND = not detected. Values are expressed as relative peak area (%) = 100 x (peak area of individual VOC / total peak area of identified VOCs). Results expressed as mean values (n = 2). Means followed by different letter in the same row showed significant difference ($p < 0.05$) within the whole sample population by Duncan's test

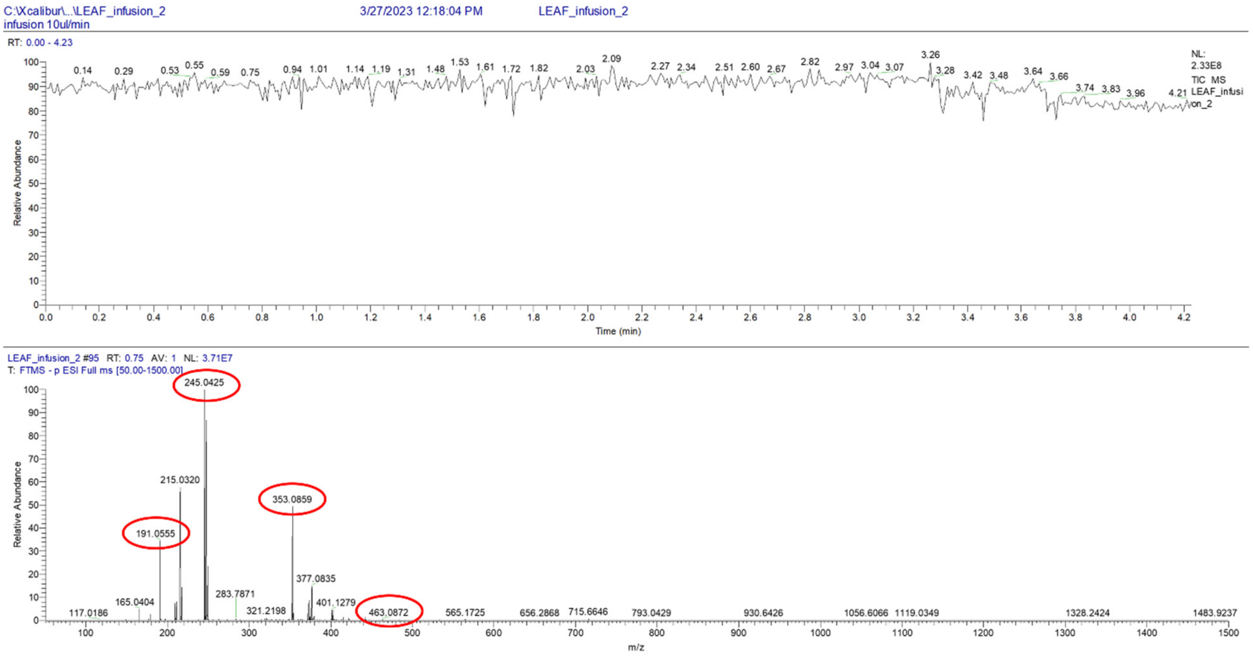

Figure S1. High-resolution mass spectrum of avocado leaf extract.

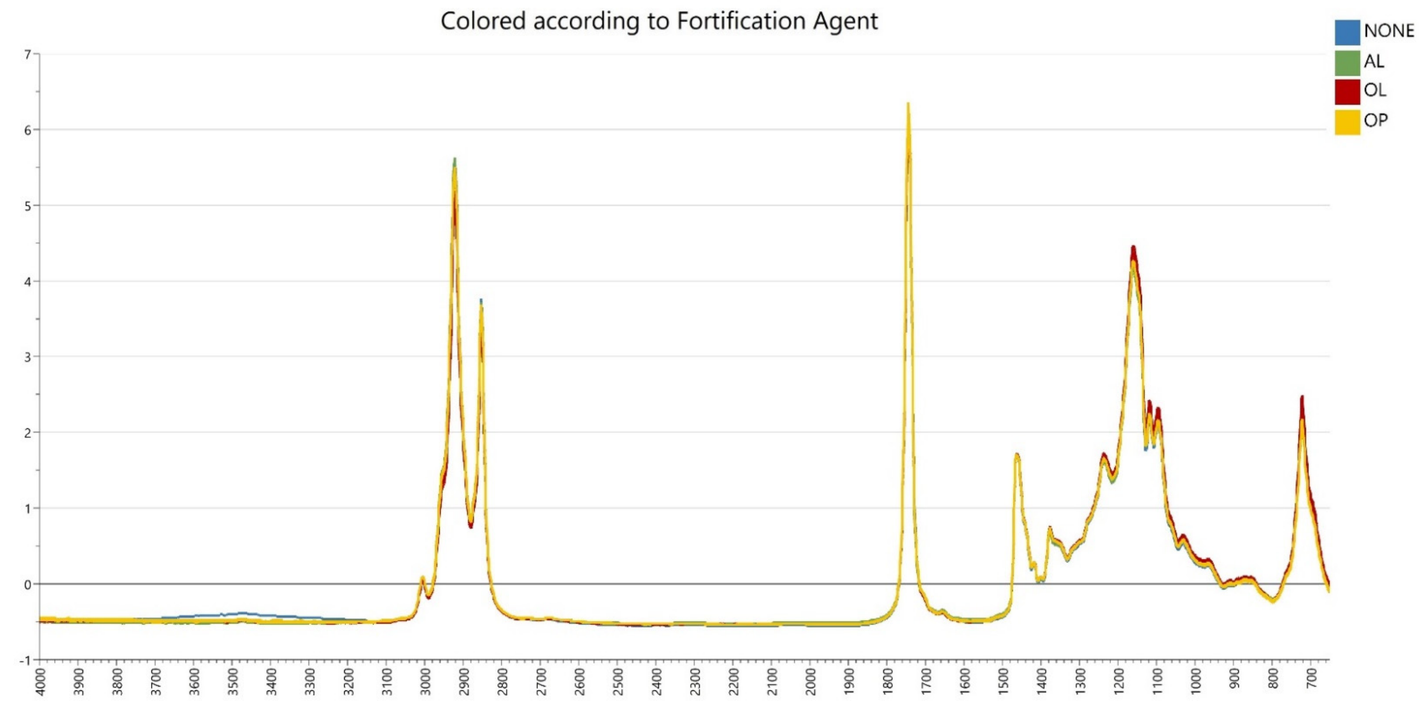

Figure S2. ATR-FT-IR spectra of the studied control (none) and enriched virgin avocado oils (AL, OL, OP).

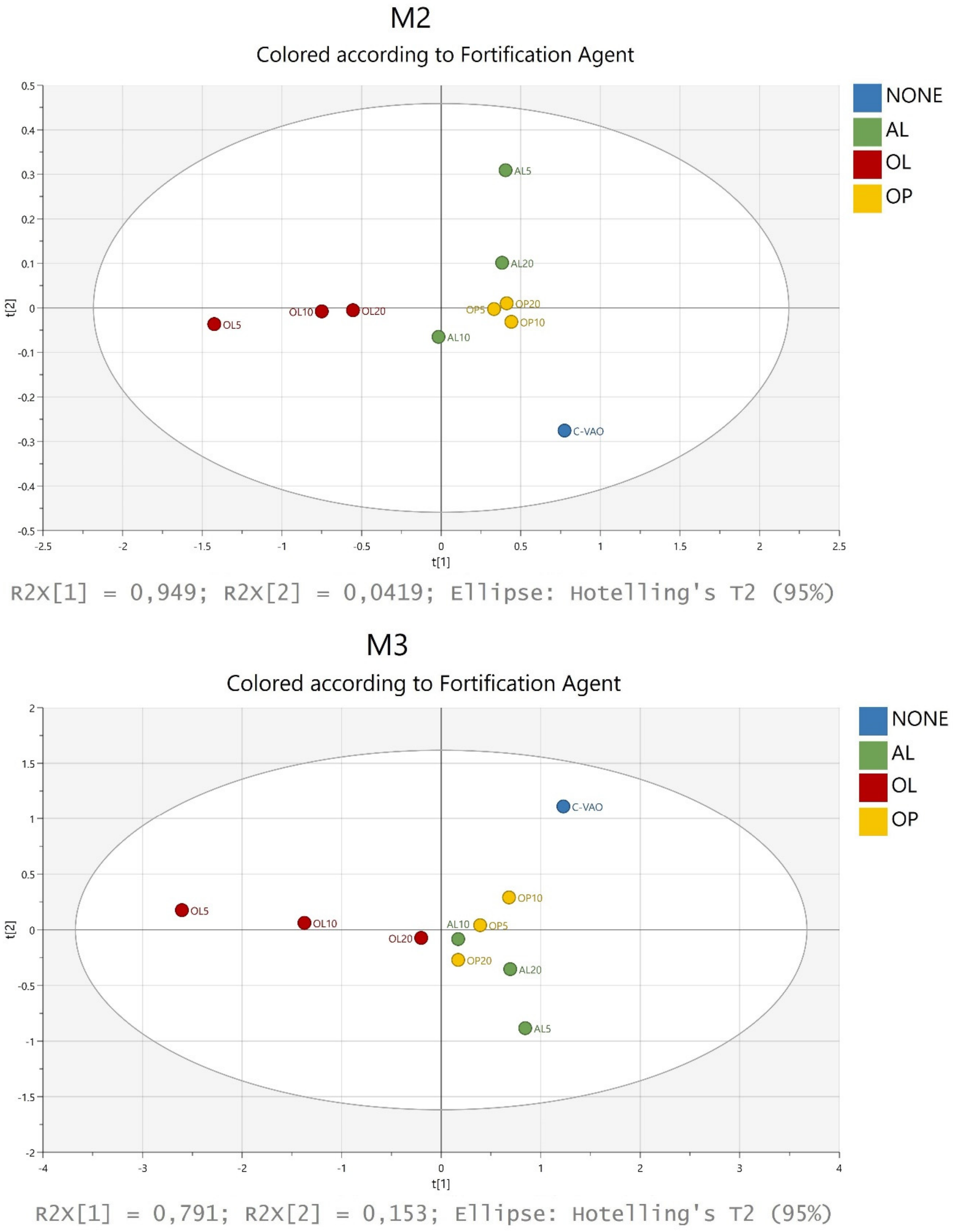

Figure S3. Scattered t1/t2 score plots of the studied control and enriched virgin avocado oils.

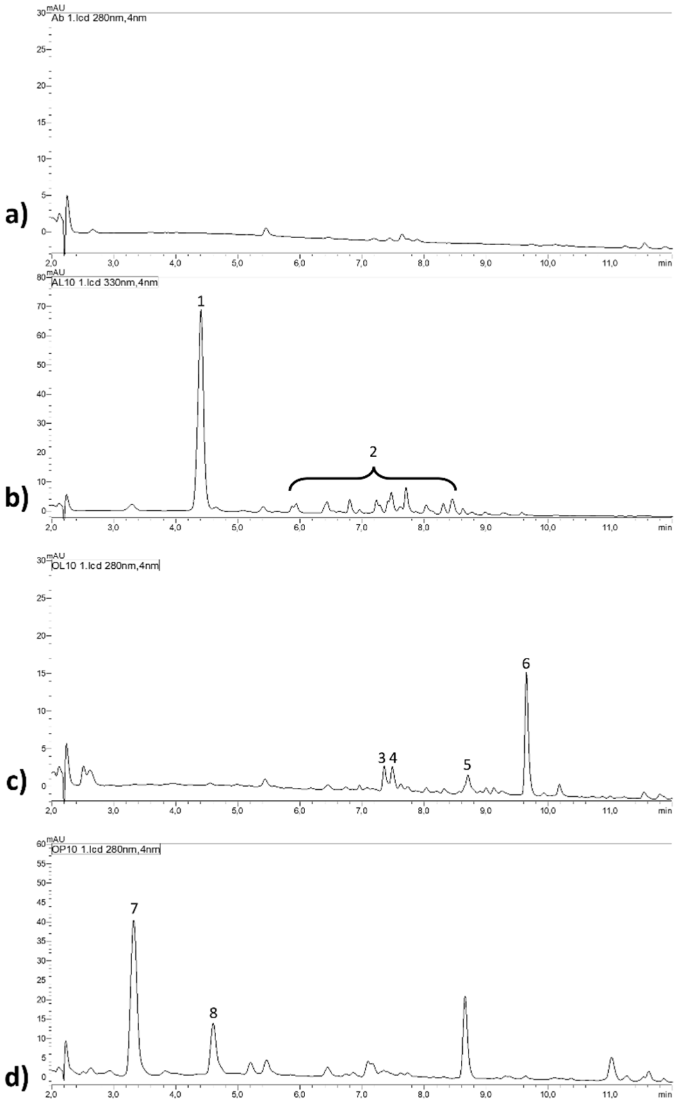

Figure S4. HPLC-DAD chromatograms of the studied control (a) and enriched virgin avocado oils with avocado leaves (b), olive leaves (c), and olive pomace (d). 1: Chlorogenic acid; 2, 5: flavonoids; 3: verbascoside; 4: luteolin-7-O-glucoside; 6: oleuropein; 7: hydroxytyrosol; 8: tyrosol.

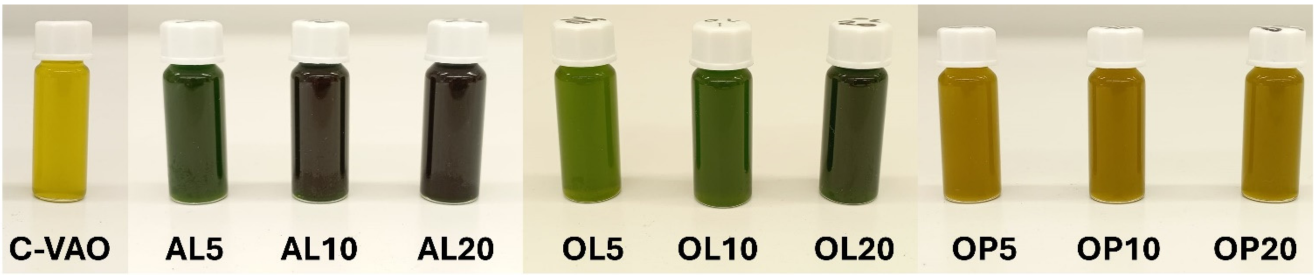

Figure S5. Pictures of the control and enriched virgin avocado oils. C-VAO = freshly-extracted virgin avocado oil, AL5-AL10-AL20, OL5-OL10-OL20 and OP5-OP10-OP20 represent C-VAO enriched with avocado leaves, olive leaves and olive pomace, respectively, at 5, 10 and 20% (w/w) employing ultrasound assisted maceration
